# Long-Term Observations of Epibenthic Fish Zonation in the Deep Northern Gulf of Mexico

**DOI:** 10.1371/journal.pone.0046707

**Published:** 2012-10-03

**Authors:** Chih-Lin Wei, Gilbert T. Rowe, Richard L. Haedrich, Gregory S. Boland

**Affiliations:** 1 Ocean Science Centre, Memorial University of Newfoundland, St. John’s, Canada; 2 Department of Marine Biology, Texas A & M University at Galveston, Galveston, Texas, United States of America; 3 Department of Biology, Memorial University of Newfoundland, St. John’s, Canada; 4 Branch of Environmental Sciences, Bureau of Ocean Energy Management, United States Department of Interior, Herndon, Virginia, United States of America; Heriot-Watt University, United Kingdom

## Abstract

A total of 172 bottom trawl/skimmer samples (183 to 3655-m depth) from three deep-sea studies, *R/V Alaminos* cruises (1964–1973), Northern Gulf of Mexico Continental Slope (NGoMCS) study (1983–1985) and Deep Gulf of Mexico Benthos (DGoMB) program (2000 to 2002), were compiled to examine temporal and large-scale changes in epibenthic fish species composition. Based on percent species shared among samples, faunal groups (≥10% species shared) consistently reoccurred over time on the shelf-break (ca. 200 m), upper-slope (ca. 300 to 500 m) and upper-to-mid slope (ca. 500 to 1500 m) depths. These similar depth groups also merged when the three studies were pooled together, suggesting that there has been no large-scale temporal change in depth zonation on the upper section of the continental margin. Permutational multivariate analysis of variance (PERMANOVA) also detected no significant species changes on the limited sites and areas that have been revisited across the studies (P>0.05). Based on the ordination of the species shared among samples, species replacement was a continuum along a depth or macrobenthos biomass gradient. Despite the well-known, close, negative relationship between water depth and macrofaunal biomass, the fish species changed more rapidly at depth shallower than 1,000 m, but the rate of change was surprisingly slow at the highest macrofaunal biomass (>100 mg C m^−2^), suggesting that the composition of epibenthic fishes was not altered in response to the extremely high macrofaunal biomass in the upper Mississippi and De Soto Submarine Canyons. An alternative is that the pattern of fish species turnover is related to the decline in macrofaunal biomass, the presumptive prey of the fish, along the depth gradient.

## Introduction

Rapid changes in faunal composition down the continental margin, or bathymetric faunal zonation, has been postulated to result from declining availability of particulate organic carbon (POC) delivered to the benthos [1], [2], [3]. Multiple biological and physical factors, such as competition, predation, temperature, and hydrostatic pressure, also contribute to the zonation pattern; however, they are often linked to each other and correlated with water depth [4], [5], [6]. It has been well established that the distribution of soft-bottom assemblages are zoned with depth in the deep ocean, usually as distinct narrow bands parallel to the isobaths [7], [8], [9], [10], [11]. Variation in faunal constituents or zonal boundaries, on the other hand, can also occur across isobaths, presumably related to the horizontal variability in physical parameters or productivity gradients within a geographic area [12], [13], [14]. While the spatial distribution and composition of benthic assemblages has been widely studied, large-scale temporal changes in presence or absence of the non-commercial, deep-sea species are not so clear, largely due to the scarcity of long-term data and potential bias associated with sampling techniques.

Nevertheless, the best available long time-series studies are in the abyssal NE Pacific [15] and in the NE Atlantic [16], where apparent temporal changes in taxa or species composition have been observed and linked to long-term, climate-driven variations in surface production and the export flux of particulate organic carbon (POC) to the seafloor [17], [18], [19], [20], [21]. While the temporal changes in invertebrate infauna and epifauna assemblages in the abyssal NE Pacific and NE Atlantic have been confirmed by these studies, the long-term changes in epibenthic fish assemblages were only apparent if the changes in population abundance were considered in the analysis [22], [23]. Based on these observations, it might be possible to infer that the temporal faunal changes may extend to areas outside of these long-time stations experiencing the same climate forcing or with similar oceanographic conditions, and thus the pattern of faunal zonation may be altered at contemporary time scales. Unfortunately, large-scale studies to test this speculation have not occurred due to the expense of long-term deep-sea research. Problems associated with consistent taxonomic identifications between historical studies and use of different sampling and analysis methods also impede temporal comparisons of species composition for the small, diverse metazoan infauna and epibenthic fishes.

In contrast to strong seasonal and inter-annual climate forcing at the long-term sites of the NE Pacific and NE Atlantic [21], the Gulf of Mexico (GoM) receives relatively constant energy supplies [24], [25], with hydrographic properties in the deepwater being constant in the past 30 to 40 years [26]. The surface phytoplankton biomass on the continental slope displays well-defined seasonal cycles [27] with little inter-annual variability observed within the northern GoM [28]. On the seafloor, the overall levels and the rates of declining benthic macrofauna biomass with depth (potential prey for epibenthic fishes) were also comparable between large-scale surveys in the 1980’s and in the 2000’s [29]. However, like any other continental margin ecosystem, the northern GoM has been subjected to substantial anthropogenic pressures (e.g. climate change, commercial shrimp trawling, coastal hypoxia, and oil and gas activities). These processes could potentially influence the distribution and occurrence of deep-sea epibenthic fishes.

In this study, we compared the zonation patterns of epibenthic fish assemblages from three large deep-sea surveys in the northern GoM: 1) R/V *Alaminos* sampling from 1964 to 1973 [30], 2) Northern Gulf of Mexico Continental Slope (NGoMCS) survey from 1983 to 1985 [31], [32], and 3) Deep Gulf of Mexico Benthos (DGoMB) program from 2000 to 2002 [33], [34]. Here, the term “zonation” was adopted to conveniently explain and visualize large-scale patterns. In order to establish a consistent criterion among studies, a cut-off of 10% species shared was used as a standard to define fish faunal zones. The objective is to detect potential long-term and large-scale changes in epibenthic fish species composition along the depth contours. Deep-sea macrobenthos biomass is known to decline exponentially with water depths due to declining quantity and quality of particulate organic carbon (POC) flux arriving at the seafloor [1], [3], [35]; hence, possible drivers behind any temporal or spatial changes in fish species composition were examined using depth and macrobenthos biomass as a proxy for resource availability.

## Materials and Methods

A direct comparison of the fish zonation among the three studies is difficult due to numerous spatial and temporal “gaps” across the database; hence, alternative approaches were utilized in this analysis. 1) The zonation pattern was examined individually for each dataset of different sampling time as well as for the three datasets pooled together based on the same criteria (at least 10% of shared species among zones). Here, we looked for large-scale patterns (such as depth zonation) to determine whether these patterns were consistent among studies and, at the same time, in accord with the zonal patterns from the pooled data. 2) In a limited number of areas and sampling sites, the historical sites were revisited in close proximity or at the exact locations. These samples were then compared directly across different studies. Both approaches were employed to cross-verify the zonation patterns among studies and examine potential temporal variation of fish species composition.

**Figure 1:**
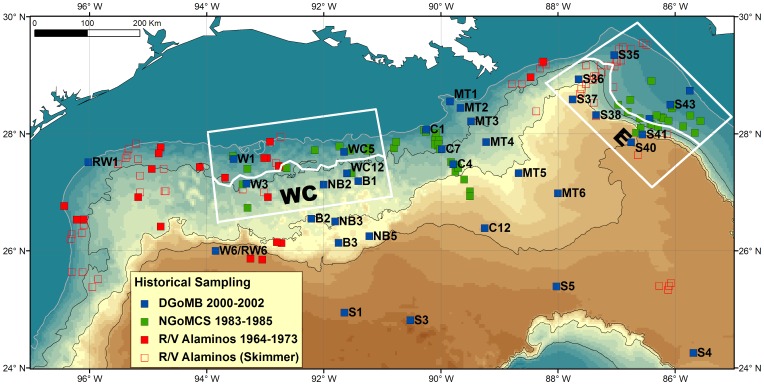
Historical sampling of deep-sea epibenthic fishes in the northern GoM. The selected areas, WC and E, were used to test the null hypotheses across three studies (color symbols) and two different depth intervals (separated by white lines in middle of the boxes). The solid symbols show the otter trawl sampling locations and the open symbols indicate the benthic skimmer sampling. The color gradients reflect the depth change from shallow to deep. The gray line indicates 200-m isobath. The black lines indicate 1,000-m isobaths. The station names give the DGoMB sampling sites.

Species presence/absence data for epibenthic fishes were obtained from the R/V *Alaminos*, NGoMCS, and DGoMB databases (Fig. 1, Table S1). A 20-m otter trawl with 76-mm stretch mesh and 25-mm cod-end mesh was used during the *Alaminos* cruises [30]. The towing time varied from 30 minutes at shallow depths to 3 hours at depths below 3,000 m. In addition to the trawl net, a 3-m gap benthic skimmer [36] was also employed on the seafloor at a speed between 2 to 4 knots for approximately 1 hour. The skimmer had a welded steel frame and wide gape with vertical and horizontal constriction in the midsection. The design was to promote central flow while providing clam pockets in the cod end to protect specimens. Pequegnat et al. [36] suggested that the skimmer collects large organisms, on, in, and above the bottom and is rugged and hydrodynamically contoured for rapid descent and fast towing for long periods of time without clogging; hence, Pequegnat et al. [36] claimed the skimmer to be an ideal sampling device for the fast moving megafauna. A total of 136 species was recorded in 80 trawls/skimmers spanning 183 to 3,365-m depth. The NGoMCS study used a 9-m swept width semi-balloon otter trawl with 38-mm stretch mesh and 13-mm cod-end mesh. The trawl was towed at a speed of 1 to 3 knots for approximately 1 hour at stations shallower than 1,300-m depth and two or more hours at deeper stations. A total of 123 species was recorded in 55 trawls from depths of 329 to 2,858 m. During the DGoMB study, a 10-m swept width semi-balloon otter trawl with 64-mm stretch mesh and 25-mm cod-end mesh was used to sample 37 locations at depths of 188 to 3,655 m. The trawl was towed at a speed of 1 to 3 knots from approximately 30 minutes at stations shallower than 1,000 m and up to 1.75 hours on the abyssal plain. A total of 152 species was recovered from the surveys.

During the NGoMCS and DGoMB study, benthic macrofauna were sampled with a 0.06 and 0.2-m^2^ GOMEX box corer [37] respectively at the same locations of bottom trawling. Macrofauna density was estimated from specimens retained on a 300-µm sieve. For selected samples, the body size of each specimen was measured using an ocular micrometer with appropriate morphometric formulae based on the animal body shape. The total biomass was estimated by multiplying the abundance with mean weight of major taxonomic groups. Macrofauna biomass from DGoMB and NGoMCS sampling are available in Table S1. Detailed sampling methods are available in Wei et al. [29].

Occurrences of fish species from three deep-sea surveys were cross-verified with the scientific names in FishBase [38] and then compiled into a single table that includes 261 species that were sampled in 172 bottom trawl/skimmer samples (Table S2, Table S3). The sample-by-species table was converted to Sørensen’s similarity matrix using the formula, QS = 2C/(A + B), where A and B are the number of species in the 2 compared samples and C is the number of species shared by the 2 samples [39]. With presence/absence data, the Sørensen’s index is equivalent to the commonly-used Bray-Curtis similarity [40] for quantitative data [41]. In order to obtain a clear dendrogram structure and obviate the influence of species that occur only once, a subset of 159 species with >1 occurrence (Table S2) was retained to calculate Sørensen’s inter-sample similarities and group-average cluster analysis [41]. The fish faunal zones (with relatively homogeneous species composition) were identified based on the prerequisite of significant clusters (SIMPROF test, P<0.05) with at least 10% of the species shared among the samples [42]. Characteristic species were identified as those with the highest occurrence within each zone.

With the exception of the cluster analysis, all multivariate analyses throughout this paper were based on the Sørensen’s similarity matrix converted from the full species list. The faunal affinity between the samples was examined by non-metric multi-dimensional scaling (MDS) represented by relative distances on a two dimensional plane [41]. The MDS axis explained most of the variation in fish species composition and was plotted against water depth or macrofaunal biomass to examine their relationships with the proxies of food availability. Because of the strong correlation between the macrofaunal biomass and depth, we used their empirical relationship, log_10_ biomass (mg C m^−2^) = 2.21−0.28×depth (km) (R^2^ = 0.72, derived from Wei et al. [29]), to examine the effect of spurious correlations. This was done by comparing the observed and simulated macrofaunal biomass (converted from depth and the above equation) against the first axis of MDS ordination; similar relationships would suggest strong spurious correlation between the patterns of fish species composition along the depth and macrofaunal biomass gradients. Spearman’s rank correlations of the fish resemblance matrix with inter-sample differences of sampling depth or macrofaunal biomass were examined using RELATE (or Mantel) test [41].

Unfortunately, during the entire course of GoM deepwater sampling, few locations have been revisited. Only the west central (WC) and east (E) areas of the northern GoM have been sampled roughly in the same proximity across all three studies (enclosed by white boxes in Fig. 1); therefore, a two-way mixed model permutational multivariate analysis of variance (PERMANOVA) [43] was conducted on these areas to test the null hypotheses that there was no change in fish species composition across the three different sampling times (fixed factor). The random factor used two depth intervals separated by 900 and 840-m isobaths in the WC and E areas respectively (solid lines within the boxes, Fig. 1). The multiple comparisons employed a low alpha level to avoid the Type 1 error (α = 2%, Bonfferoni correction) [41].

The DGoMB (2000–2002) study repeated the NGoMCS (1983–1985) sampling at Stations W1, W3, WC5, WC12, C1, C7 and C4 (Fig. 1). The DGoMB program also sampled in the proximity of the NGoMCS and *Alaminos* sites at Stations S41, S42 and S43, as well as Stations S35, S36, S37 and S38, respectively (Fig. 1). Since no replication was available for these locations, a randomized complete block (RCB) PERMANOVA was employed to test for temporal variation in species composition. The blocking factor used different sampling sites along the selected transect. To increase sample size for the PERMANOVA tests, Stations W1, W3, WC5 and WC12 were combined as a single transect before conducting the analysis.

The two-way mixed model PERMANOVA was also conducted on pooled data across studies of different sampling times (fixed factor). The random factor used 4 depth intervals to divide the pooled data into identical size of samples. Because most of the locations were only sampled once across the three studies, this pooled PERMANOVA test neglected potential spatial variability and assumed that all the samples were collected from a single depth transect.

The multivariate and GIS analyses used PRIMER 6 & PERMANOVA+ and ESRI® ArcMap™ 9.2. Violin plot used R 2.15.0 [44] and R packages “vioplot” and “sm” [45], [46].

## Results

### R/V *Alaminos* Sampling from 1964 to 1973

**Figure 2:**
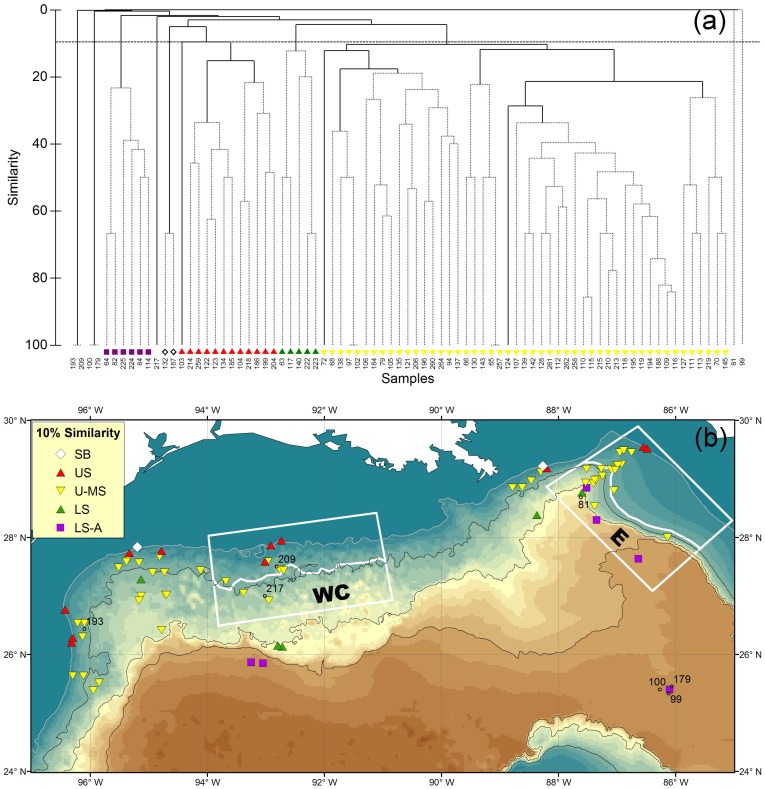
Epibenthic fish species composition and faunal zonation during the R/V *Alaminos* cruises from 1964 to 1973. (a) Group-average cluster analysis on inter-sample Sørensen’s similarities. The solid lines indicate significant structure (SIMPROF test, P<5%). The horizontal dashed line shows 10% similarity. (b) Distribution of the fish faunal zones with at least 10% of faunal similarity. “SB” denotes Shelf-Break Group. “US” denotes Upper-Slope Group. “U-MS” denotes Upper-to-Mid Slope Group. “LS” denotes Lower-Slope Group. “LS-A” denotes Lower-Slope-to-Abyssal Group. The same symbols are used in Fig. 2a and 2b.

Group-average cluster analysis and SIMPROF test on inter-sample Sørensen’s similarities suggested 7 significant groups (P<0.05, Fig. 2a). Koefoed’s smooth-head (*Bathytroctes macrolepis*) and *Nezumia cyrano* were the only species found in trawl no. 100 and 179 as well as no. 193 and 209 respectively and had limited distributions in other trawls (Fig. 2a); hence, these two cluster groups has almost no similarities with the rest of the samples and were 100% similar within the groups. Among the cluster groups with higher affinity, the two shallowest sites shared 66.7% of species while the rest of the groups shared 10.3 to 23.3% of species (Fig. 2a). Shelf-Break (SB) and Upper-Slope (US) Groups extended from depths of 183 to 210 m and 183 to 538 m, respectively (Fig. 2b). Upper-to-Mid-Slope (U-MS) Group occupied part of the upper slope and most of the mid slope from depths of 379 to 1,829 m. Lower-Slope (LS) Group had one station on the mid slope at 1,134-m depth and covered the lower slope between depths of 1,829 and 2,140 m. Low-Slope-to-Abyssal (LS-A) Group represented the deepest sampling from 2,103 to 3,287-m depth. A comparison between the distribution of cluster groups (Fig. 2b) and the locations of otter trawl (solid red squares, Fig. 1) and benthic skimmer samples (empty red squares) showed that the homogeneous groups were mostly defined by the depth contours rather than by the gear types. In other word, the otter trawl and benthic skimmer were capable of recovering similar species.

### Northern Gulf of Mexico Continental Slope (NGoMCS) Study from 1983 to 1985

**Figure 3:**
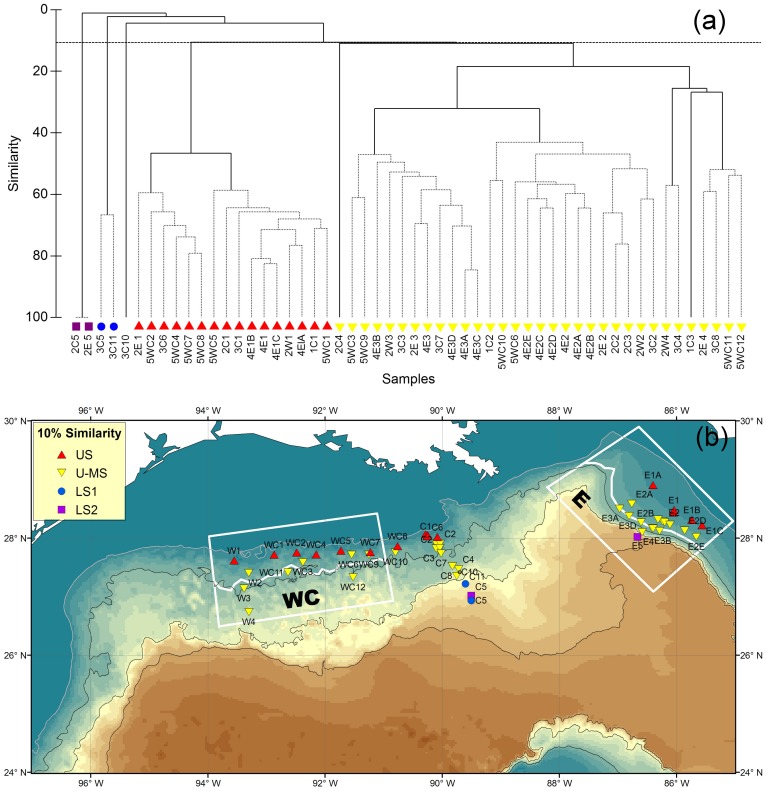
Epibenthic fish species composition and faunal zonation during the NGoMCS study from 1983 to 1985. (a) Group-average cluster analysis on inter-sample Sørensen’s similarities. The solid lines indicate significant structure (SIMPROF test, P<5%). The horizontal dashed line shows 10% similarity. (b) Distribution of the fish faunal zones with at least 10% of faunal similarity. “US” denotes Upper-Slope Group. “U-MS” denotes Upper-to-Mid-Slope Group. “LS1” denotes Lower-Slope Group 1. “LS2” denotes Lower-Slope Group 2. The same symbols are used in Fig. 3a and 3b.

A total of 4 significant groups (P<0.05) were identified by cluster analysis and SIMPROF (Fig. 3a). Upper-Slope (US) and Upper-to-Mid Slope (U-MS) Groups were separated at 10% of Sørensen’s similarity with the sampling depths extending from 329 to 552 m and 603 to 1,510 m, respectively (Fig. 3b). The 2 deepest groups, Lower-Slope 1 (LS1) and Lower-Slope 2 (LS2), occupied depths from 2,074 to 2,504 m and from 2,401 to 2,858 m, respectively.

### Deep Gulf of Mexico Benthos (DGoMB) Program from 2000 to 2002

**Figure 4:**
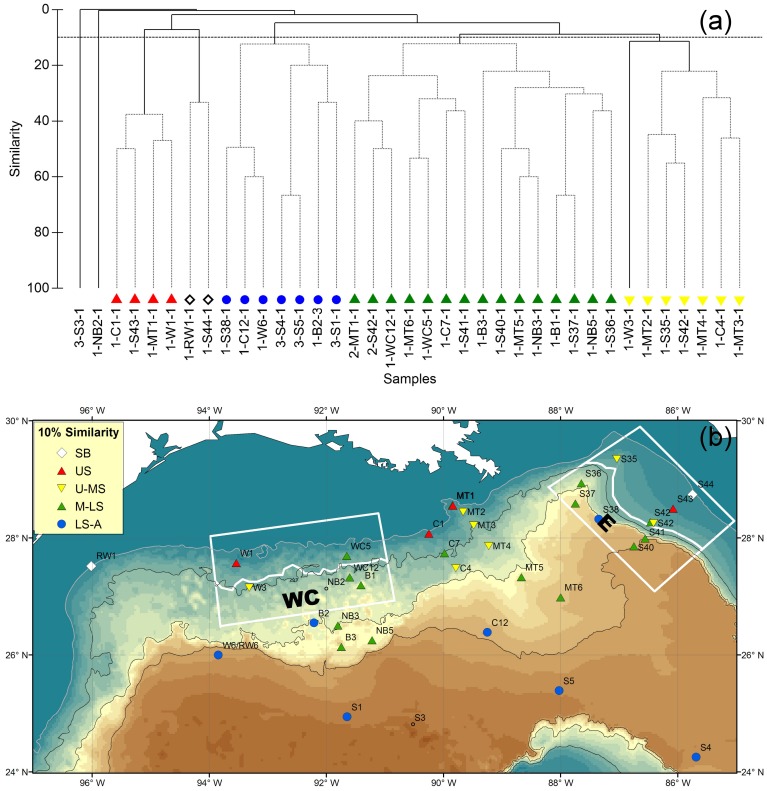
Epibenthic fish species composition and faunal zonation during the DGoMB study from 2000 to 2002. (a) Group-average cluster analysis on inter-sample Sørensen’s similarities. The solid lines indicate significant structure (SIMPROF test, P<5%). The horizontal dashed line shows 10% similarity. (b) Distribution of the fish faunal zones with at least 10% of faunal similarity. “SB” denotes Shelf-Break Group. “US” denotes Upper-Slope Group. “U-MS” denotes Upper-to-Mid-Slope Group. “M-LS” denotes Mid-to-Lower-Slope Group. “LS-A” denotes Lower-Slope-to-Abyssal Group. The same symbols are used in Fig. 4a and 4b.

Cluster analysis with SIMPROF suggested 5 significant groups (P<0.05) sharing at least 10% to 36.6% of species (Fig. 4a). Shelf-Break (SB) Group included the two shallowest sites at depths of 188 and 213 m (Fig. 4b). Upper-Slope (US) Group occurred between 325 and 461-m depth. Upper-to-Mid-Slope (U-MS) Group extended from depths of 670 m to 1,369 m. Mid-to-Lower-Slope (M-LS) Group covered the largest sampling area, including shallower distribution at Station WC5, WC12 and C7 between depths of 758 and 1,100 m and the other deep sites extending from 1,784 to 3,010-m depth. It is worth noting that the epibenthic fish assemblages in WC5 and WC12 were more closely resembled the deeper assemblages from the lower slope, as opposed to the typical upper slope assemblages at the other two sites to the west (W1 and W3). Lower-Slope-to-Abyssal (LS-A) Group was distributed exclusively from 2,608 to 3,590-m depth.

### Hypothesis Testing

Depth had significant effects on fish species composition of the E (PERMANOVA, depth, P = 0.001, Table 1a) and WC areas (depth, P = 0.001, Table 1b); however, only a marginal temporal effect was detected in the E area (time, P = 0.082, Table 1a). In the WC area, a significant interaction was also detected between the sampling time and depth block (time × depth, P = 0.03, Table 1b); hence, pairwise comparisons were conducted within each of the two depth blocks and confirmed no statistical difference between all pairs of the sampling times (P>0.02, Bonfferoni correction). Except for W1, W3, WC5, and WC12 (PERMANOVA, site, P = 0.178, Table 1c), the sampling site (along a depth transect) had significant effects on species composition (site, P≤0.01, Table 1d to 1f). No statistical temporal effect was detected between the historical and revisited sites in all transects (PERMANOVA, time, P = 0.076 to 0.445, Table 1c to f).

**Table 1:** Permutational multivariate analysis of variance (PERMANOVA) on epibenthic fish species composition among 3 deep-sea surveys conducted between 1964 and 2002 in the northern GoM.

Source	df	SS	MS	Pseudo-F	P
**(a) E area (across 3 studies)**					
Time	2	19213	9607	2.04	0.082
Depth	1	11539	11539	3.17	0.001
Time × Depth	2	9411	4706	1.29	0.107
Error	46	167260	3636		
**(b) WC area (across 3 studies)**					
Time	2	11883	5942	1.19	0.337
Depth	1	10969	10969	3.20	0.001
Time × Depth	2	10005	5002	1.46	0.03
Error	22	75347	3425		
**(c) W1, W3, WC5 & WC12 (NGoMCS vs. DGoMB)**					
Time	1	5029	5029	1.54	0.246
Site	1	14551	4850	1.49	0.178
Error	3	9779	3260		
**(d) C1, C7 & C4 (NGoMCS vs. DGoMB)**					
Time	1	2774	2774	1.06	0.445
Site	2	16031	8016	3.08	0.007
Error	5	13033	2607		
					
**(e) S41, S42 & S43 (NGoMCS vs. DGoMB)**					
Time	1	5168	5168	1.89	0.076
Site	2	15433	7717	2.83	0.009
Error	5	13649	2730		
**(f) S35, S36, S37 & S38 (** ***Alaminos*** ** vs. DGoMB)**					
Time	1	5268	5268	1.93	0.103
Depth	3	16699	5566	2.04	0.01
Error	4	10901	2725		

Two-way mixed model PERMANOVA were employed for (a) the east and (b) the west central areas which roughly overlap for all 3 surveys (Fig. 1). The random factor used two depth intervals separated by the 840-m depth contour in the east area and 900-m depth contour in the west central area. For a limited number of sites, the most current DGoMB study repeated the historical NGoMCS or R/V *Alaminos* sampling; hence, randomized complete block (RCB) PERMANOVA was conducted on (c) Station W1, W3, WC5 and WC12, (d) Station C1, C7 and C4, (e) Station S41, S42 and S43, and (f) S35, S36, S37 and S38 to examine the temporal variation on fish species composition. The blocking factor for the RCB PERMANOVA used different sites along the selected transects.

### Pooled Data from Year 1964 to 2002

**Figure 5:**
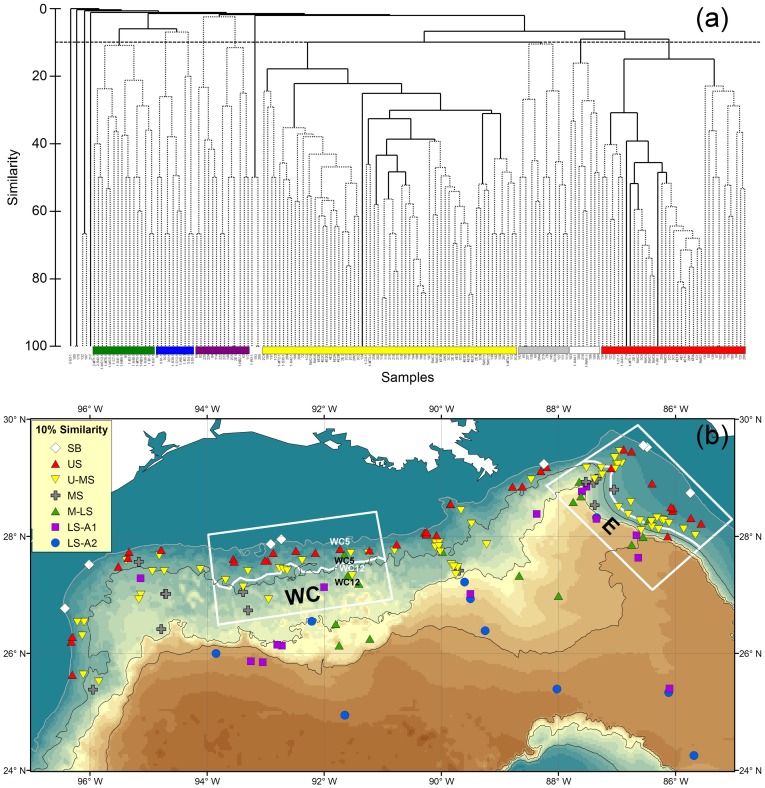
Epibenthic fish species composition and faunal zonation for the pooled data from 1964 to 2002. (a) Group-average cluster analysis on inter-sample Sørensen’s similarities. The solid lines indicate significant structure (SIMPROF test, P<5%). The horizontal dashed line shows 10% similarity. (b) Distribution of the fish faunal zones with at least 10% of faunal similarity. “SB” denotes Shelf-Break Group. “US” denotes Upper-Slope Group. “U-MS” denotes Upper-Slope-to-Mid-Slope Group. “MS” denotes Mid-Slope Group. “M-LS” denotes Mid-to-Lower-Slope Group. “LS-A1” denotes Lower-Slope-to-Abyssal Group 1. “LS-A2” denotes Lower-Slope-to-Abyssal Group 2. The same symbols are used in Fig. 5a and 5b.

A total of 10 significant groups (P<0.05) were identified from the pooled data based on cluster analysis and SIMPROF (Fig. 5a). Seven clusters with the largest area coverage were plotted on Fig. 5b. The majority of these cluster groups shared at least 10 to 17.1% of species; however, in order to meet the prerequisite of significant cluster structure (SIMPROF test, P<0.05), Lower-Slope-to-Abyssal 1 (LS-A1) and Lower-Slope-to-Abyssal 2 (LS-A2) Groups shared only 2.4 and 8.1% of the species, respectively (Fig. 5a & b).

**Figure 6:**
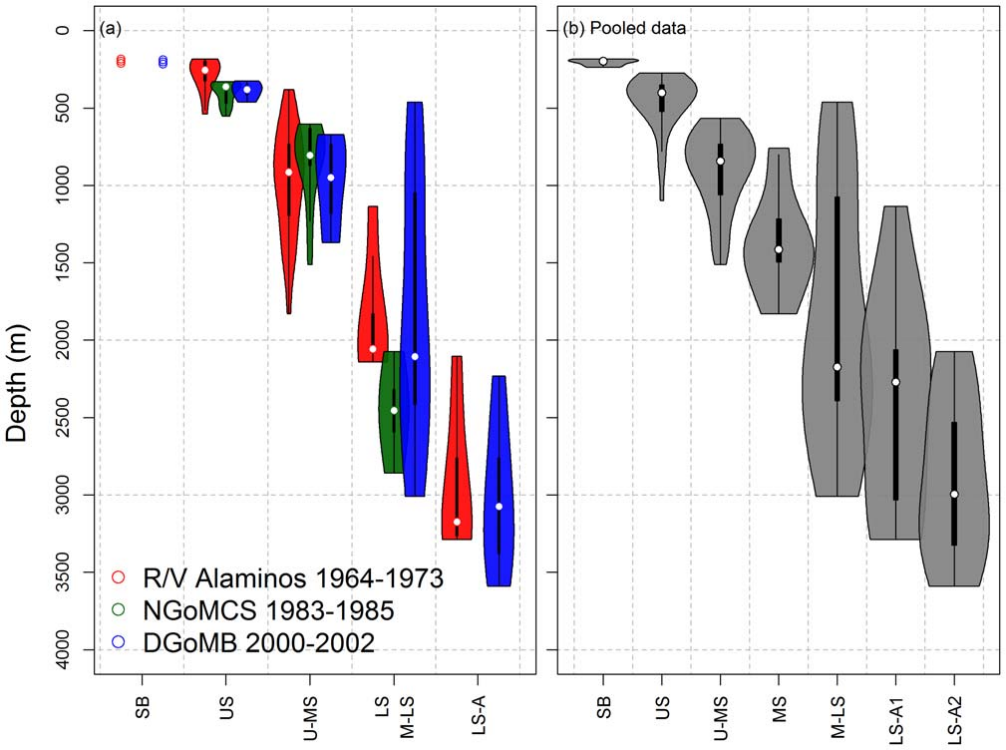
Violin plots of sampling depths for homogenous faunal groups in (a) R/V *Alaminos*, NGoMCS, and DGoMB studies, and (b) pooled data of all three studies. The violin plot is a combination of box plot and kernel density plot, which started with a standard box plot (with minimum, lower quartile, median, upper quartile, and maximum depth values) and then added rotating kernel density plots to each side of the box plot. When the sampling depths were equal or less than three observations, the raw depth values were shown directly.

Depth distributions of Shelf-Break (SB), Upper-Slope (US) and Upper-to-Mid Slope (U-MS) Groups were consistent across three sampling times at approximately 200 to 300 m, 300 to 500m, 500 to 1500 m, respectively (Fig. 6a). This recurring pattern also matched the SB, US, and U-MS zones based on the cluster analysis of pooled data (Fig. 6b). Moreover, although the SB, US, and U-MS zones had only a few overlapping stations across the studies (Fig. 2b, 3b and 4b), they merged into their respective depth zones in the pooled analysis (Fig. 5b). This evidence suggests that large-scale temporal change of depth zonation had not occurred on the upper section of the continental slope because the homogenous groups (in the pooled analysis) would have been separated by study rather than by depth, if any changes had occurred during the three sampling times. Nevertheless, the cluster analyses assigned the same locations (WC5 and WC12) from the NGoMCS and DGoMB sampling to the shallow (white letters) and deep cluster groups (black letters, Fig. 5b) respectively, supporting a potential small-scale shift in species composition toward resembling lower slope assemblages. In deeper water, similar depth zones across the three studies did not merge in the pooled analysis. For example, the Mid-to-Lower Slope (M-LS) and Lower-Slope-to-Abyssal (LS-A2) Groups in the pooled data (Fig. 6b) corresponded mostly to M-LS and LS-A Groups of the DGoMB sampling (blue color, Fig. 6a) respectively. The LS-A1 Group from the pooled analysis (Fig. 6b) was mostly derived from the LS and LS-A Groups in the R/V *Alaminos* sampling (red color, Fig. 6a).

**Figure 7:**
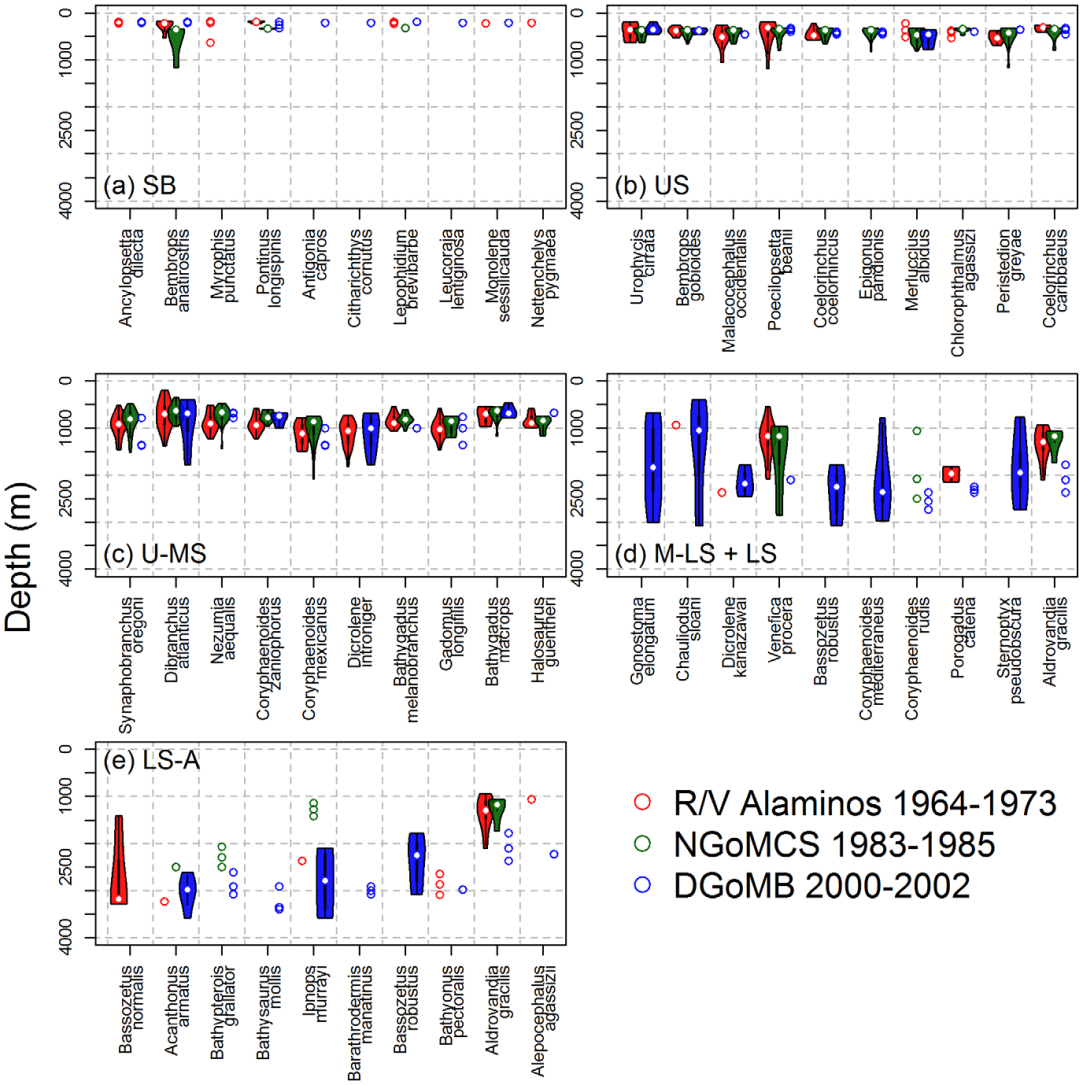
Violin plots of sampling depths for the top-10 most common species (with highest occurrence) from (a) Shelf Break, (b) Upper Slope, (c) Upper-to-Mid Slope, (d) Mid-to-Lower and Lower Slope, and (d) Lower-Slope-to-Abyssal Groups. Colors indicate the studies of different sampling times. The violin plot is a combination of box plot and kernel density plot, which started with a standard box plot (with minimum, lower quartile, median, upper quartile, and maximum depth values) and then added rotating kernel density plots to each side of the box plot. When the sampling depths were equal or less than three observations, the raw depth values were shown directly.

Distribution of the top-10 most common species (with highest occurrence) in similar depth groups are shown in Figure 7 and Table S4. Except for the duckbill flathead (*Bembrops anatirostris*), the common species of Shelf-Break (SB) Groups were mostly restricted to the edge of continental shelf (Fig. 7a). It should be noted that the NGoMCS sampling started from the upper slope (316 m) and did not have a SB zone; hence, some of the common SB species appeared to occur deeper in the NGoMCS (green) than in the *Alaminos* (red) or DGoMB sampling (blue). In the Upper-Slope (US, Fig. 7b) and Upper-to-Mid-Slope Groups (U-MS, Fig. 7c), most of the common species occurred across the three sampling times with their distributions being consistently at ∼400 and ∼1000-m depths, respectively. In the Mid-to-Lower Slope (M-LS) and Low Slope Groups (LS, Fig. 7d) as well as the Lower Slope-to-Abyssal Group (LS-A, Fig. 7d), the top-10 most common species, however, were mostly derived from the DGoMB sampling (blue), because the DGoMB focused more on deepwater trawling than the other studies. Interestingly, most of the depth groups were characterized by different common species (Fig. 7). Only the robust assfish (*Bassozetus robustus*) and *Aldrovandia gracilis* were the most common species in both the M-LS + LS (Fig. 7d) and LS-A Groups (Fig. 7e). These restricted distributions suggested strong depth dependence for the common species.

**Figure 8:**
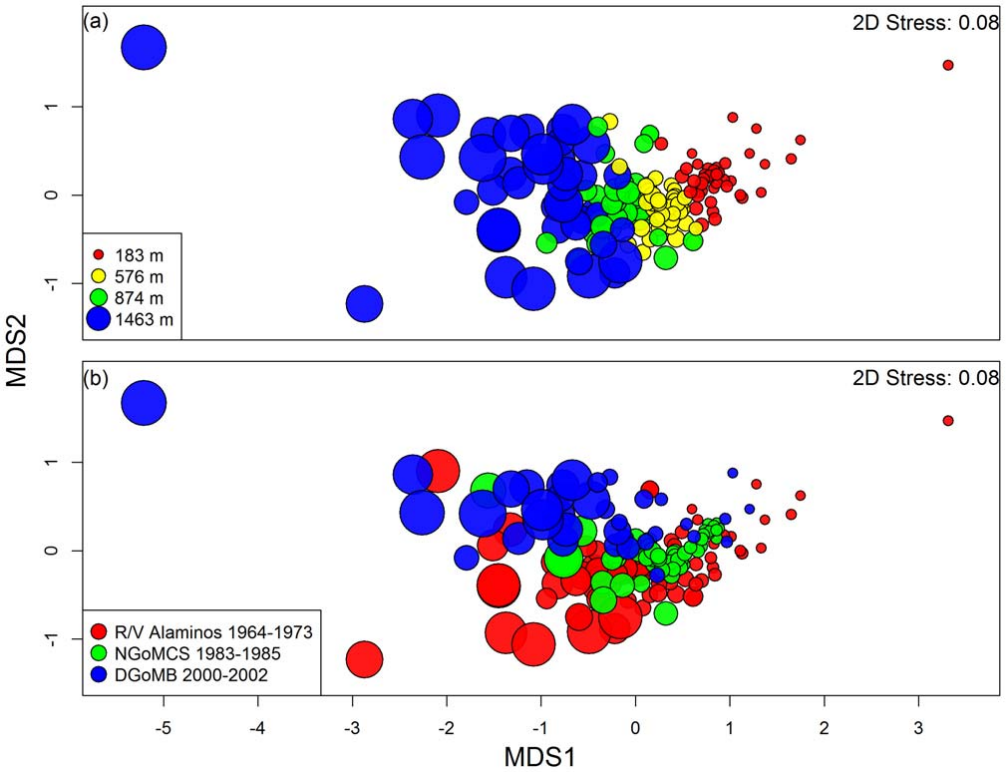
Non-metric multi-dimensional scaling (MDS) on inter-sample Sørensen’s similarities of pooled data. The distances between samples represent relative dissimilarities in species composition. (a) Symbol sizes show relative water depths with colors indicating four depth intervals with equivalent numbers of samples. (b) Symbol sizes show relative depth with colors indicating three studies of different sampling times.

**Figure 9:**
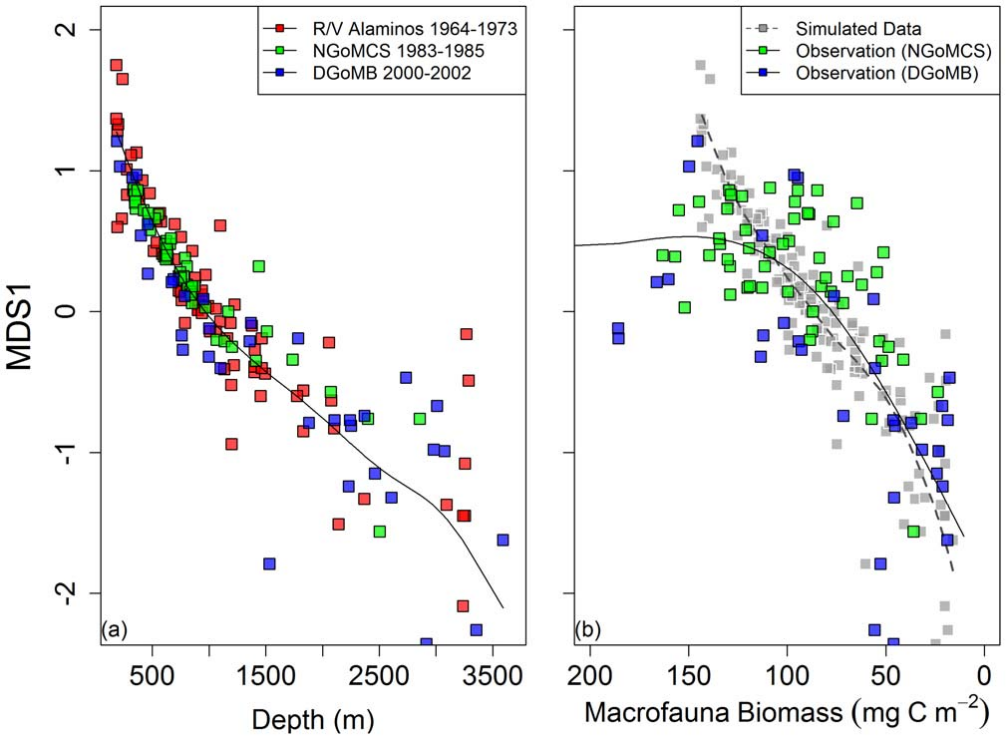
The x-axis of the non-metric multi-dimensional scaling (MDS1) plotted against (a) depth and (b) total macrofaunal biomass. The MDS1 represents species composition of epibenthic fishes in multivariate space. The trend lines show the MDS1 as smooth spline functions of depth or macrofaunal biomass. The depth values in Fig. 9a were converted to simulated macrofaunal biomass using an empirical equation from the northern GoM, Log_10_ biomass (mg C m^−2^) = 2.21–0.28 * depth (km) (R^2^ = 0.72, P<0.001) and then plotted against the MDS1 (Fig. 9b, gray squares). The two largest empirical biomass values (640 and 439 mg C m^−2^ at MT1) were not shown (Fig. 7b, blue squares) but had been included in the estimation of spline function. Their corresponded MDS1 values were 0.55 and 0.21, respectively.

The placement of samples on non-metric multi-dimensional scaling (MDS) illustrates a continuum of changes in fish species composition from the shelf break to the abyssal plain without distinct boundaries along the depth gradient (Fig. 8a). Studies of different sampling times, on the other hand, were overlapped on the same ordination plot (Fig. 8b). The x axis (MDS1) of the ordination appears to follow the depth gradient and depth thus contributes to most of the variation in the MDS plot (Fig. 8a). The y axis (MDS2) can roughly define the three studies but contributes considerably less to the ordination (Fig. 8b). A two-way cross PERMANOVA (across the 4 depth blocks, Fig. 8a) suggested significant temporal (F_2,160_ = 2.26, P = 0.012) and depth (F_3, 160_ = 10.53, P = 0.001) effects but also identified a significant interaction between sampling time and depth block (F_6, 160_ = 2.06, P = 0.001). This is not unexpected because the NGoMCS study (green symbols, Fig. 8b) sampled a smaller depth range than the *R/V Alaminos* (red symbols) and DGoMB studies (blue symbols). When the shallowest and deepest depth blocks were removed, no statistical temporal difference was found among the three studies (F_2, 80_ = 2.89, P = 0.098) but the depth effect was still significant over the two middle depth blocks (Fig. 8a, F_1, 80_ = 5.7, P = 0.001). The rate of change for the MDS1 with depth was more rapid on the upper slope (<1,000-m depth) than the lower slope and abyssal plain (Fig. 9a). The similar MDS1-depth relationships had occurred across the three studies of different sampling times (red, green, and blue symbols). Nevertheless, the MDS1 changed more rapidly at the lower end (<100 mg C m^−2^) than the higher end of the macrofauna biomass (Fig. 9b, green/blue symbols and solid line). The macrobenthos biomass was not available for the R/V *Alaminos* study, but similar slow rates of changes in MDS1 at the high macrofaunal biomass were observed for the NGoMCS (green symbols) and DGoMB studies (blue symbols). Simulated macrofaunal biomass-MDS1 trend (Fig. 9b, gray symbols and dashed line) was comparable to the observed trend (green/blue symbols and solid black line) at the lower biomass (<100 mg C m^−2^) but the relationship broke down at high biomass (>100 mg C m^−2^), suggesting that the empirical biomass-MDS1 relationship cannot be explained by a mathematic relationship between the macrofaunal biomass and depth. The fish Sørensen’s similarity matrix, however, was more tightly correlated to the water depth (RELATE, ρ = 0.69, P<0.001) than to the macrofaunal biomass (RELATE, ρ = 0.238, P<0.001).

## Discussion

Our analyses of individual studies and the pooled data agreed with previous investigations showing distinct depth zonation without noticeable horizontal faunal changes along isobaths [30], [32], [33]. On the upper continental slope, evidence such as 1) no statistical difference in species composition among the revisited sites, areas, and depth blocks; 2) consistent depth distributions of homogeneous groups and common species; 3) merging of similar depth groups in the pooled analysis; and 4) overlapped placements of different studies on the ordination map, suggested that there was no large-scale temporal change of depth zonation across different times of sampling. Nevertheless, PERMANOVA over the entire depth ranges did find evidence of temporal effect in fish species composition. It is worth noting that this statistical evidence was a combination of temporal and spatial effects, because the majority of sampling sites were not repeated across the studies. The lack of shelf break and abyssal samples during the NGoMCS sampling and the focus of deep and unexplored areas during the DGoMB study could also contribute to substantial bias in our two-way cross design (e.g. interaction between sampling time and depth blocks). If we consider that the sampling patterns within the depth blocks were not the same across the three studies, a two-way nested PERMANOVA (depth blocks nested within studies) would suggest no temporal difference in species composition (F_2, 171_ = 0.88, P = 0.583). Nevertheless, cluster analysis identified a potential small-scale shift of species composition (toward more resemble the deepwater communities) in the upper slope of west central (WC) area. This was not detected by the direct statistical tests, probably because the assemblage shift was only observed at WC5 and WC12 and its effect might be diluted by the other two revisited sites in the WC area (W1 and W3).

Even though the lower-slope and lower-slope-to-abyssal zones reoccurred across three studies, these similar depth zones did not fall into the same cluster groups in the analysis of pooled data. The observed pattern usually reflected the faunal zones from either the R/V *Alaminos* (1964–1973) or the DGoMB (2000–02) study, because the NGoMCS (1983–85) study only had two sampling sites below 2,000-m depth. The sampling on the lower slope and abyssal plain was generally scattered and the majority of the sites were only visited once during the three studies; hence, it is difficult to discern whether the affiliation of group to a specific study is due to spatial heterogeneity in species composition, temporal changes in faunal zonation, or simply the sampling gear difference. The R/V *Alaminos* studies used a combination of benthic skimmer and otter trawl while the NGoMCS and DGoMB were sampled exclusively by the otter trawl. Pooling the skimmer and otter trawl samples does not seem to affect the consistency of the zonal pattern during the R/V *Alaminos* study; however, when the fish abundance declined with depth [32], [33], the gear effect could be magnified because the skimmer may be more capable of catching agile organisms [36]. While this might be reasonable speculation, there was no evidence that the homogeneous groups were separated by gear type on the lower slope and abyssal plain during the R/V *Alaminos* study.

Since no apparent change of zonal pattern was evident among studies of different sampling times, we combined the three data sets to examine the large-scale species turnover as a function of depth, confirming a gradual, continuum change of species composition along a depth gradient [11], [14], [47]. Many species occupy overlapping ranges [10], [11], with immutable boundaries being rare; hence, the zonal pattern observed here is better described as the rate of species replacement along a habitat gradient [6], or β diversity [48], [49]. Based on Terborgh’s [50] theory of species distribution on environmental gradients, the continuum of species turnover in this study is more likely related to continuous variations with depth (temperature, pressure [51] or decline of export POC flux [14]), rather than abrupt shifts in water mass structure [52], [53] or the steep boundary at the oxygen minimum zone [54]. In the northern GoM, the variability of hydrographic properties becomes greatly reduced below depth of 800 m and their horizontal distribution was uniform below the depth of the Yucatan sill (ca. 1,500-m depth) [26], [55]. This homogeneity may contribute to the slightly slower rate of change in faunal composition (or lower β diversity) on the lower-slope and abyssal plain compared to the upper-slope depths. In deepwater, the exponential decline of export POC flux with depth was probably the main driving force for the pattern of β diversity [6], because the selection for pressure-resistant species occurs at relatively shallow depths (ca. 500 to 1,000 m) [51].

Interestingly, the discord between the simulated and observed trends in Figure 9b suggests that the changes in epibenthic fish species composition with macrofaunal biomass contradicted its relationship with depth. If the macrofaunal biomass declined exponentially with depth [1], [3], [29], [35], the rapid species replacement on the shelf edge and upper slope (above 1,000-m depths) would translate to a fast turnover at the higher macrofaunal biomass, as illustrated in Figure 9b (gray squares and dashed line). In fact, the rate of change in species composition was surprisingly low when the macrofaunal biomass was the highest (>100 mg C m^−2^). This may be biased because the deep sites (∼500–1800 m) within or in proximity to the Mississippi and De Soto Canyons had extremely high macrofaunal biomass [29]. These outliers can not be predicted by an exponential depth decay model and thus the species turnover with macrofaunal biomass (green/blue squares and black line, Fig. 9b) deviated from our expectation (gray squares and dashed line). Nevertheless, this disparity suggests that although the species replacement was continuous with the increasing macrofaunal biomass (or decreasing depths), the fish composition did not respond to the elevated biomass at the canyon associated sites. The motile epibenthic fishes may feed on a broad spectrum of benthic and pelagic prey and macrobenthos may not be their preferred prey [56], [57], [58], [59]. Some deep-sea macrourids evidently bypass benthic food web through scavenging carrion [60] or the variable prey available in canyons.

Rex [61] hypothesized that at high trophic levels, such as epibenthic fishes, the assemblage structure would be influenced more by competition, as opposed to lower trophic-level macrofauna, being affected more by predation [62]. He proposed that when competition is strong, species may repulse one another, giving rise to fewer overlapping ranges of distribution and thus more pronounced zonation along a resource gradient [6], [50]. This hypothesis poses an alternative explanation for the relationship between macrofaunal biomass (productivity) and rates of change in epibenthic fish species composition (β diversity). The macrofauna, *per se*, is not the only diet for the epibenthic fishes but it might shed some light on the overall level of export POC flux delivered to the benthos [63], [64], [65]. Conventionally, competitive exclusion is accelerated when resources are abundant and population densities are high [6], such as high macrofaunal biomass or export POC flux on the shelf edge and upper slope. This may be true to some degree for the less motile, deposit-feeding megafauna invertebrates with similar feeding guilds [7]; however, the motile epibenthic fish is not limited to specific prey items [57], [66]. Based on this hypothesis, the slow fish species replacement at the high macrofaunal biomass may be a function of reduced competition due to abundant and more variable resources near the submarine canyons.

Obviously, our interpretation of the observed ‘productivity-β diversity’ relationship is conjecture based on a few snapshots of fish assemblage structure (the MDS plots). Biological interactions are complicated and likely act together with environmental heterogeneity to shape the pattern of faunal zonation or β diversity [67]. Perhaps any temporal changes were overwhelmed by the immense depth variation in our large-scale analyses. Nevertheless, the presence of deep-sea epibenthic fish species in the northern GoM, at least on the upper slope, provides no tangible evidence that fish assemblages have undergone any major changes in the past 40 years.

## Supporting Information

Table S1 **Average latitude, longitude, and depth of epibenthic fish sampling locations in the northern Gulf of Mexico.** “Trawl” denotes the unique sample ID shared between Table S1 and S3. “Biom” denotes macrofauna biomass (mg C m^−2^) collected using Box Corer along with the trawl sampling. Unit: Depth (m), Area (hectare). Alaminos cruises were conducted between 1964 and 1973; however, the exact date and sampling area for each sample was not available (NA).
(DOC)

Table S2 **Species list of deep-sea epibenthic fishes during** ***Alaminos*** **, NGoMCS, and DGoMB surveys in the northern Gulf of Mexico.** Only species with valid scientific names were listed. “Code” denotes the unique species ID shared between Table S2 and S3. The code was list based on the alphabetical order of species names. Species name, family, environment, and common name were based on the Fishbase. “Occurrence” indicates the number of times (trawls) that the specific species was recovered. “Depth” indicates the minimum and maximum occurrence depths.
(DOC)

Table S3 **Occurrence and abundance of deep-sea epibenthic fishes during the** ***Alaminos*** **, NGoMCS, and DGoMB surveys in the northern Gulf of Mexico.** “Trawl” denotes the unique sample ID shared between Table S1 and S3. “Code” denotes the unique species ID shared between Table S2 and S3. “N” denotes number of specimen recovered from each trawl sample.
(DOC)

Table S4 **The characteristic epibenthic fish species in the northern Gulf of Mexico.** The top-10 species with the highest occurrence were listed for each faunal group based on the cluster analysis of pooled data. “Code” corresponds to the species code in Table S2. “Occur” denotes number of occurrence and “% Occur” denotes percentage of occurrence in specific faunal zones.
(DOC)
