# Supplementary material for: Long-Term Observations of Epibenthic Fish Zonation in the Deep Northern Gulf of Mexico
Source: PLoS One. 2012 Oct 3;7(10):e46707. doi: 10.1371/journal.pone.0046707 (PMC3463567; doi:10.1371/journal.pone.0046707)
Supplement: Table S2 — Species list of deep-sea epibenthic fishes during Alaminos , NGoMCS, and DGoMB surveys in the northern Gulf of Mexico. Only species with valid scientific names were listed. “Code” denotes the unique species ID shared between Table S2 and S3. The code was list based on the alphabetical order of species names. Species name, family, environment, and common name were based on the Fishbase. “Occurrence” indicates the number of times (trawls) that the specific species was recovered. “Depth” indicates the minimum and maximum occurrence depths. (DOC) [file pone.0046707.s002.doc]

Table S2. Species list of deep-sea epibenthic fishes during *Alaminos*, NGoMCS, and DGoMB surveys in the northern Gulf of Mexico. Only species with valid scientific names were listed. “Code” denotes the unique species ID shared between Table S2 and S3. The code was list based on the alphabetical order of species names. Species name, family, environment, and common name were based on the Fishbase. “Occurrence” indicates the number of times (trawls) that the specific species was recovered. “Depth” indicates the minimum and maximum occurrence depths.

| **Code** | **Specie Name** | **Family** | **Environment** | **Common Name** | **Occurrence** | **Depth** |
| --- | --- | --- | --- | --- | --- | --- |
|  |  |  |  |  |  |  |
| 238 | *Synagrops bellus* | Acropomatidae | Bathydemersal | Blackmouth bass | 12 | 325-663 |
| 239 | *Synagrops spinosus* | Acropomatidae | Bathydemersal | Keelcheek bass | 6 | 188-354 |
| 261 | *Zenopsis conchifer* | Acropomatidae | Benthopelagic | Silvery John dory | 2 | 325-345 |
| 5 | *Alepocephalus agassizii* | Alepocephalidae | Bathydemersal | Agassiz' slickhead | 2 | 1061-2230 |
| 6 | *Alepocephalus productus* | Alepocephalidae | Bathydemersal | Smalleye smooth-head | 3 | 1784-2369 |
| 38 | *Bathytroctes macrolepis* | Alepocephalidae | Bathypelagic | Koefoed's smooth-head | 5 | 1006-3255 |
| 39 | *Bathytroctes microlepis* | Alepocephalidae | Bathypelagic | Smallscale smooth-head | 2 | 1359-3590 |
| 65 | *Conocara macropterum* | Alepocephalidae | Bathypelagic | Longfin smooth-head | 9 | 1006-1829 |
| 141 | *Leptoderma macrops* | Alepocephalidae | Bathydemersal | Grenadier smooth-head | 4 | 686-871 |
| 163 | *Narcetes stomias* | Alepocephalidae | Bathypelagic | Blackhead salmon | 4 | 1784-2608 |
| 217 | *Rinoctes nasutus* | Alepocephalidae | Bathypelagic | Abyssal smooth-head | 2 | 1170-3655 |
| 218 | *Rouleina maderensis* | Alepocephalidae | Bathypelagic | Madeiran smooth-head | 1 | 2105 |
| 242 | *Talismania antillarum* | Alepocephalidae | Bathypelagic | Antillean smooth-head | 1 | 758 |
| 7 | *Anacanthobatis folirostris* | Anacanthobatidae | Bathydemersal | Leaf-nose leg skate | 3 | 351-475 |
| 9 | *Anoplogaster cornuta* | Anoplogastridae | Bathypelagic | Common fangtooth | 1 | 2243 |
| 22 | *Barathronus bicolor* | Aphyonidae | Bathypelagic |  | 13 | 576-3267 |
| 222 | *Sciadonus galatheae* | Aphyonidae | Bathydemersal |  | 1 | 629 |
| 223 | *Sciadonus pedicellaris* | Aphyonidae | Bathydemersal |  | 1 | 628 |
| 14 | *Argentina striata* | Argentinidae | Bathypelagic | Striated argentine | 8 | 229-369 |
| 85 | *Dolicholagus longirostris* | Bathylagidae | Bathypelagic | Longsnout blacksmelt | 1 | 1784 |
| 158 | *Monolene sessilicauda* | Bothidae | Bathydemersal | Deepwater flounder | 2 | 216-229 |
| 248 | *Trichopsetta ventralis* | Bothidae | Demersal | Sash flounder | 3 | 188-237 |
| 47 | *Bregmaceros atlanticus* | Bregmacerotidae | Pelagic | Antenna codlet | 1 | 384 |
| 84 | *Diplacanthopoma brachysoma* | Bythitidae | Bathydemersal |  | 5 | 576-752 |
| 219 | *Saccogaster maculata* | Bythitidae | Bathydemersal |  | 1 | 361 |
| 220 | *Saccogaster staigeri* | Bythitidae | Bathydemersal |  | 1 | 354 |
| 100 | *Foetorepus agassizii* | Callionymidae | Bathydemersal | Spotfin dragonet | 2 | 192-476 |
| 10 | *Antigonia capros* | Caproidae | Demersal | Deepbody boarfish | 1 | 216 |
| 49 | *Caranx hippos* | Carangidae | Reef | Crevalle jack | 1 | 325 |
| 78 | *Decapterus punctatus* | Carangidae | Reef | Round scad | 1 | 786 |
| 148 | *Macroramphosus scolopax* | Centriscidae | Demersal | Longspine snipefish | 2 | 192-329 |
| 53 | *Chaunax pictus* | Chaunacidae | Bathydemersal | Pink frogmouth | 25 | 350-840 |
| 54 | *Chaunax suttkusi* | Chaunacidae | Bathydemersal |  | 1 | 670 |
| 136 | *Kali indica* | Chiasmodontidae | Bathypelagic |  | 1 | 758 |
| 209 | *Pseudoscopelus sp.* | Chiasmodontidae | Bathypelagic |  | 1 | 1100 |
| 55 | *Chimaera monstrosa* | Chimaeridae | Bathydemersal | Rabbit fish | 2 | 636-906 |
| 128 | *Hydrolagus alberti* | Chimaeridae | Bathydemersal |  | 3 | 538-1369 |
| 129 | *Hydrolagus colliei* | Chimaeridae | Demersal | Spotted ratfish | 1 | 696 |
| 130 | *Hydrolagus mirabilis* | Chimaeridae | Bathydemersal | Large-eyed rabbitfish | 2 | 853-1002 |
| 56 | *Chlorophthalmus agassizi* | Chlorophthalmidae | Bathydemersal | Shortnose greeneye | 17 | 329-538 |
| 57 | *Chlorophthalmus chalybeius* | Chlorophthalmidae | Bathydemersal |  | 2 | 311-412 |
| 182 | *Parasudis truculenta* | Chlorophthalmidae | Demersal | Longnose greeneye | 15 | 274-1170 |
| 63 | *Coloconger meadi* | Colocongridae | Bathydemersal |  | 2 | 670-785 |
| 2 | *Acromycter perturbator* | Congridae | Bathydemersal |  | 3 | 965-1170 |
| 18 | *Ariosoma balearicum* | Congridae | Reef | Bandtooth conger | 1 | 3655 |
| 41 | *Bathyuroconger vicinus* | Congridae | Bathypelagic | Large-toothed conger | 3 | 850-1136 |
| 64 | *Conger oceanicus* | Congridae | Demersal | American conger | 1 | 585 |
| 208 | *Pseudophichthys splendens* | Congridae | Bathydemersal | Purplemouthed conger | 14 | 229-871 |
| 215 | *Rhynchoconger flavus* | Congridae | Demersal | Yellow conger | 1 | 183 |
| 216 | *Rhynchoconger guppyi* | Congridae | Demersal |  | 1 | 351 |
| 249 | *Uroconger syringinus* | Congridae | Demersal | Threadtail conger | 1 | 192 |
| 256 | *Xenomystax atrarius* | Congridae | Bathydemersal | Deepwater conger | 1 | 192 |
| 257 | *Xenomystax bidentatus* | Congridae | Demersal |  | 1 | 229 |
| 236 | *Symphurus marginatus* | Cynoglossidae | Bathydemersal | Margined tonguefish | 13 | 351-752 |
| 237 | *Symphurus piger* | Cynoglossidae | Bathydemersal | Deepwater tonguefish | 2 | 384-476 |
| 93 | *Etmopterus gracilispinis* | Dalatiidae | Bathypelagic | Broadbanded lanternshark | 2 | 475-632 |
| 94 | *Etmopterus pusillus* | Dalatiidae | Benthopelagic | Smooth lanternshark | 2 | 628-636 |
| 95 | *Etmopterus schultzi* | Dalatiidae | Bathydemersal | Fringefin lanternshark | 19 | 400-853 |
| 96 | *Etmopterus spinax* | Dalatiidae | Bathydemersal | Velvet belly | 2 | 538-585 |
| 97 | *Etmopterus virens* | Dalatiidae | Bathydemersal | Green lanternshark | 3 | 465-479 |
| 48 | *Bufoceratias wedli* | Diceratiidae | Bathypelagic |  | 1 | 1002 |
| 213 | *Remora brachyptera* | Echeneidae | Pelagic | Spearfish remora | 1 | 2230 |
| 87 | *Epigonus denticulatus* | Epigonidae | Bathydemersal | Pencil cardinal | 2 | 465-522 |
| 88 | *Epigonus macrops* | Epigonidae | Bathydemersal | Luminous deepsea cardinalfish | 1 | 828 |
| 89 | *Epigonus occidentalis* | Epigonidae | Bathydemersal | Western deepsea cardinalfish | 3 | 369-850 |
| 90 | *Epigonus pandionis* | Epigonidae | Bathydemersal | Bigeye | 19 | 329-828 |
| 107 | *Gibberichthys pumilus* | Gibberichthyidae | Bathypelagic | Gibberfish | 1 | 758 |
| 108 | *Gigantura indica* | Giganturidae | Bathypelagic | Telescopefish | 1 | 2608 |
| 75 | *Cyclothone alba* | Gonostomatidae | Bathypelagic | Bristlemouth | 1 | 2735 |
| 76 | *Cyclothone pallida* | Gonostomatidae | Bathypelagic | Tan bristlemouth | 1 | 998 |
| 110 | *Gonostoma atlanticum* | Gonostomatidae | Bathypelagic | Atlantic fangjaw | 1 | 998 |
| 111 | *Gonostoma elongatum* | Gonostomatidae | Bathypelagic | Elongated bristlemouth fish | 14 | 670-3010 |
| 152 | *Manducus maderensis* | Gonostomatidae | Bathypelagic |  | 1 | 346 |
| 3 | *Aldrovandia affinis* | Halosauridae | Benthopelagic | Gilbert's halosaurid fish | 13 | 788-1784 |
| 4 | *Aldrovandia gracilis* | Halosauridae | Bathypelagic |  | 20 | 937-2369 |
| 112 | *Halosaurus guentheri* | Halosauridae | Bathypelagic |  | 23 | 585-1170 |
| 113 | *Halosaurus ovenii* | Halosauridae | Bathydemersal |  | 5 | 603-1002 |
| 118 | *Heptranchias perlo* | Hexanchidae | Bathydemersal | Sharpnose sevengill shark | 1 | 354 |
| 127 | *Howella sherborni* | Howellidae | Bathypelagic | Sherborn's pelagic bass | 1 | 3655 |
| 31 | *Bathypterois bigelowi* | Ipnopidae | Bathydemersal |  | 2 | 625-834 |
| 32 | *Bathypterois grallator* | Ipnopidae | Bathydemersal | Tripodfish | 5 | 2074-3075 |
| 33 | *Bathypterois longipes* | Ipnopidae | Bathydemersal |  | 3 | 732-3287 |
| 34 | *Bathypterois phenax* | Ipnopidae | Bathydemersal | Blackfin spiderfish | 2 | 1170-3010 |
| 35 | *Bathypterois quadrifilis* | Ipnopidae | Bathydemersal |  | 16 | 937-2077 |
| 36 | *Bathypterois viridensis* | Ipnopidae | Bathydemersal |  | 4 | 628-1170 |
| 40 | *Bathytyphlops sewelli* | Ipnopidae | Bathydemersal |  | 1 | 2243 |
| 135 | *Ipnops murrayi* | Ipnopidae | Bathydemersal |  | 7 | 1136-3590 |
| 79 | *Decodon puellaris* | Labridae | Reef | Red hogfish | 1 | 192 |
| 144 | *Lophiodes monodi* | Lophiidae | Bathydemersal |  | 2 | 367-622 |
| 145 | *Lophius gastrophysus* | Lophiidae | Bathydemersal | Blackfin goosefish | 7 | 350-479 |
| 207 | *Pristipomoides aquilonaris* | Lutjanidae | Demersal | Wenchman | 3 | 192-229 |
| 27 | *Bathygadus favosus* | Macrouridae | Bathydemersal |  | 7 | 937-1225 |
| 28 | *Bathygadus macrops* | Macrouridae | Bathypelagic | Bullseye grenadier | 27 | 461-1170 |
| 29 | *Bathygadus melanobranchus* | Macrouridae | Bathydemersal | Vaillant's grenadier | 31 | 538-1064 |
| 50 | *Cetonurus globiceps* | Macrouridae | Bathypelagic | Globehead grenadier | 2 | 1189-1376 |
| 60 | *Coelorinchus caribbaeus* | Macrouridae | Bathydemersal | Blackfin grenadier | 15 | 274-786 |
| 61 | *Coelorinchus coelorhincus* | Macrouridae | Benthopelagic | Hollowsnout grenadier | 27 | 229-663 |
| 62 | *Coelorinchus occa* | Macrouridae | Bathydemersal | Swordsnout grenadier | 4 | 751-1061 |
| 66 | *Coryphaenoides alateralis* | Macrouridae | Bathydemersal |  | 1 | 950 |
| 67 | *Coryphaenoides carapinus* | Macrouridae | Bathydemersal | Carapine grenadier | 1 | 1097 |
| 68 | *Coryphaenoides carminifer* | Macrouridae | Bathypelagic | Carmine grenadier | 1 | 636 |
| 69 | *Coryphaenoides mediterraneus* | Macrouridae | Bathypelagic | Mediterranean grenadier | 6 | 785-2980 |
| 70 | *Coryphaenoides mexicanus* | Macrouridae | Bathydemersal | Mexican grenadier | 33 | 751-2074 |
| 71 | *Coryphaenoides rudis* | Macrouridae | Bathypelagic | Rudis rattail | 5 | 1064-2735 |
| 72 | *Coryphaenoides zaniophorus* | Macrouridae | Bathydemersal | Thickbeard grenadier | 33 | 585-1225 |
| 103 | *Gadomus arcuatus* | Macrouridae | Bathypelagic | Doublethread grenadier | 13 | 670-1225 |
| 104 | *Gadomus dispar* | Macrouridae | Bathydemersal |  | 2 | 663-871 |
| 105 | *Gadomus longifilis* | Macrouridae | Bathypelagic | Treadfin grenadier | 30 | 585-1463 |
| 106 | *Gadomus macrops* | Macrouridae | Bathypelagic | *Gadomus arcuatus*? | 1 | 785 |
| 132 | *Hymenocephalus billsam* | Macrouridae | Bathydemersal |  | 1 | 400 |
| 133 | *Hymenocephalus italicus* | Macrouridae | Benthopelagic | Glasshead grenadier | 15 | 274-576 |
| 137 | *Kuronezumia bubonis* | Macrouridae | Benthopelagic | Bulbous rattail | 1 | 632 |
| 149 | *Malacocephalus laevis* | Macrouridae | Bathydemersal | Softhead grenadier | 1 | 400 |
| 150 | *Malacocephalus occidentalis* | Macrouridae | Bathydemersal | Western softhead grenadier | 29 | 274-1061 |
| 170 | *Nezumia aequalis* | Macrouridae | Benthopelagic | Common Atlantic grenadier | 51 | 475-1438 |
| 171 | *Nezumia atlantica* | Macrouridae | Bathydemersal | Western Atlantic grenadier | 10 | 576-812 |
| 172 | *Nezumia bairdii* | Macrouridae | Benthopelagic | Marlin-spike grenadier | 3 | 937-1161 |
| 173 | *Nezumia cyrano* | Macrouridae | Bathypelagic |  | 19 | 461-1359 |
| 174 | *Nezumia sclerorhynchus* | Macrouridae | Benthopelagic | Roughtip grenadier | 1 | 476 |
| 175 | *Nezumia suilla* | Macrouridae | Bathydemersal |  | 5 | 828-1359 |
| 228 | *Sphagemacrurus grenadae* | Macrouridae | Bathypelagic | Pugnose grenadier | 7 | 937-1463 |
| 229 | *Squalogadus modificatus* | Macrouridae | Bathypelagic | Tadpole whiptail | 7 | 937-1359 |
| 244 | *Trachonurus sulcatus* | Macrouridae | Bathypelagic | Bristly grenadier | 1 | 785 |
| 245 | *Trachonurus villosus* | Macrouridae | Bathypelagic | Furry whiptail | 6 | 732-1217 |
| 254 | *Ventrifossa macrogon* | Macrouridae | Bathydemersal |  | 1 | 359 |
| 255 | *Ventrifossa macropogon* | Macrouridae | Bathydemersal | Longbeard grenadier | 1 | 522 |
| 202 | *Poromitra crassiceps* | Melamphaidae | Bathypelagic | Crested bigscale | 1 | 2105 |
| 203 | *Poromitra megalops* | Melamphaidae | Bathypelagic |  | 1 | 1002 |
| 153 | *Melanonus zugmayeri* | Melanonidae | Bathypelagic | Arrowtail | 2 | 851-1203 |
| 155 | *Merluccius albidus* | Merlucciidae | Bathydemersal | Offshore silver hake | 28 | 229-812 |
| 156 | *Merluccius bilinearis* | Merlucciidae | Demersal | Silver hake | 6 | 192-636 |
| 231 | *Steindachneria argentea* | Merlucciidae | Bathydemersal | Luminous hake | 14 | 183-461 |
| 157 | *Microstoma microstoma* | Microstomatidae | Bathypelagic | Slender argentine | 1 | 998 |
| 101 | *Gadella imberbis* | Moridae | Benthopelagic | Beardless codling | 15 | 342-871 |
| 102 | *Gadella maraldi* | Moridae | Benthopelagic | Gadella | 4 | 412-696 |
| 138 | *Laemonema barbatulum* | Moridae | Benthopelagic | Shortbeard codling | 15 | 423-663 |
| 139 | *Laemonema goodebeanorum* | Moridae | Benthopelagic |  | 1 | 461 |
| 190 | *Physiculus fulvus* | Moridae | Benthopelagic | Hakeling | 2 | 199-566 |
| 191 | *Physiculus kaupi* | Moridae | Bathydemersal |  | 1 | 538 |
| 80 | *Diaphus lucidus* | Myctophidae | Bathypelagic | Spotlight lanternfish | 1 | 2230 |
| 131 | *Hygophum taaningi* | Myctophidae | Bathypelagic |  | 1 | 2980 |
| 177 | *Notoscopelus resplendens* | Myctophidae | Bathypelagic | Patchwork lampfish | 1 | 1880 |
| 91 | *Eptatretus minor* | Myxinidae | Bathydemersal |  | 1 | 465 |
| 92 | *Eptatretus springeri* | Myxinidae | Bathydemersal | Gulf hagfish | 5 | 475-775 |
| 162 | *Myxine glutinosa* | Myxinidae | Demersal | Hagfish | 1 | 851 |
| 166 | *Neoscopelus macrolepidotus* | Neoscopelidae | Bathypelagic | Large-scaled lantern fish | 3 | 752-970 |
| 167 | *Neoscopelus microchir* | Neoscopelidae | Bathypelagic | Shortfin neoscopelid | 1 | 670 |
| 124 | *Hoplunnis macrurus* | Nettastomatidae | Demersal | Freckled pike-conger | 1 | 192 |
| 125 | *Hoplunnis schmidti* | Nettastomatidae | Demersal |  | 1 | 192 |
| 126 | *Hoplunnis tenuis* | Nettastomatidae | Bathydemersal | Spotted pike-conger | 2 | 192-229 |
| 168 | *Nettastoma melanurum* | Nettastomatidae | Bathydemersal | Blackfin sorcerer | 9 | 618-851 |
| 169 | *Nettenchelys pygmaea* | Nettastomatidae | Benthopelagic | Pygmy pikeconger | 1 | 210 |
| 253 | *Venefica procera* | Nettastomatidae | Bathydemersal |  | 20 | 538-2858 |
| 176 | *Notacanthus chemnitzii* | Notacanthidae | Benthopelagic | Spiny eel | 3 | 629-751 |
| 194 | *Polyacanthonotus africanus* | Notacanthidae | Bathypelagic |  | 1 | 1217 |
| 195 | *Polyacanthonotus merretti* | Notacanthidae | Bathydemersal |  | 3 | 843-1136 |
| 81 | *Dibranchus atlanticus* | Ogcocephalidae | Bathydemersal | Atlantic batfish | 66 | 192-1784 |
| 260 | *Zalieutes mcgintyi* | Ogcocephalidae | Demersal | Tricorn batfish | 1 | 216 |
| 178 | *Oneirodes eschrichtii* | Oneirodidae | Bathypelagic | Bulbous dreamer | 1 | 937 |
| 161 | *Myrophis punctatus* | Ophichthidae | Reef | Speckled worm-eel | 3 | 183-636 |
| 1 | *Acanthonus armatus* | Ophidiidae | Bathypelagic | Bony-eared assfish | 7 | 2505-3590 |
| 20 | *Barathrites iris* | Ophidiidae | Bathydemersal |  | 1 | 3655 |
| 21 | *Barathrodemus manatinus* | Ophidiidae | Benthopelagic |  | 2 | 2915-3075 |
| 23 | *Bassogigas gillii* | Ophidiidae | Bathydemersal |  | 1 | 3010 |
| 24 | *Bassozetus normalis* | Ophidiidae | Bathydemersal |  | 6 | 1404-3287 |
| 25 | *Bassozetus robustus* | Ophidiidae | Bathydemersal | Robust assfish | 7 | 1784-3075 |
| 30 | *Bathyonus pectoralis* | Ophidiidae | Bathydemersal |  | 3 | 2651-3092 |
| 82 | *Dicrolene introniger* | Ophidiidae | Bathydemersal | Digitate cusk eel | 33 | 686-1829 |
| 83 | *Dicrolene kanazawai* | Ophidiidae | Bathydemersal |  | 7 | 1784-2460 |
| 140 | *Lepophidium brevibarbe* | Ophidiidae | Demersal | Shortbeard cusk-eel | 5 | 188-329 |
| 146 | *Luciobrotula corethromycter* | Ophidiidae | Bathydemersal |  | 4 | 625-1784 |
| 159 | *Monomitopus agassizii* | Ophidiidae | Benthopelagic |  | 18 | 752-1359 |
| 160 | *Monomitopus magnus* | Ophidiidae | Bathydemersal |  | 1 | 3355 |
| 164 | *Neobythites gilli* | Ophidiidae | Benthopelagic | Twospot brotula | 1 | 192 |
| 165 | *Neobythites marginatus* | Ophidiidae | Benthopelagic | Stripefin brotula | 1 | 538 |
| 183 | *Penopus microphthalmus* | Ophidiidae | Bathydemersal |  | 2 | 937-1359 |
| 200 | *Porogadus catena* | Ophidiidae | Bathydemersal |  | 6 | 1829-2369 |
| 201 | *Porogadus miles* | Ophidiidae | Bathydemersal | Slender cuskeel | 2 | 2243-2980 |
| 258 | *Xyelacyba myersi* | Ophidiidae | Bathydemersal | Gargoyle cusk | 4 | 1170-1829 |
| 8 | *Ancylopsetta dilecta* | Paralichthyidae | Demersal | Three-eye flounder | 4 | 188-229 |
| 58 | *Citharichthys cornutus* | Paralichthyidae | Bathydemersal | Horned whiff | 1 | 216 |
| 59 | *Citharichthys gymnorhinus* | Paralichthyidae | Demersal | Anglefin whiff | 1 | 192 |
| 181 | *Paralichthys squamilentus* | Paralichthyidae | Demersal | Broad flounder | 1 | 216 |
| 77 | *Cyttopsis roseus* | Parazenidae | Bathypelagic | Rosy dory | 2 | 325-400 |
| 43 | *Bembrops anatirostris* | Percophidae | Bathydemersal | Duckbill flathead | 14 | 183-1170 |
| 44 | *Bembrops gobioides* | Percophidae | Demersal | Goby flathead | 28 | 274-654 |
| 184 | *Peristedion ecuadorense* | Peristediidae | Bathydemersal |  | 1 | 345 |
| 185 | *Peristedion greyae* | Peristediidae | Demersal |  | 23 | 329-1170 |
| 186 | *Peristedion miniatum* | Peristediidae | Bathydemersal | Armored searobin | 6 | 216-423 |
| 187 | *Peristedion thompsoni* | Peristediidae | Bathydemersal | Rimspine searobin | 1 | 461 |
| 197 | *Polymetme corythaeola* | Phosichthyidae | Benthopelagic | Rendezvous fish | 4 | 338-752 |
| 259 | *Yarrella blackfordi* | Phosichthyidae | Bathydemersal |  | 21 | 461-1225 |
| 189 | *Phycis chesteri* | Phycidae | Benthopelagic | Longfin hake | 2 | 476-566 |
| 250 | *Urophycis cirrata* | Phycidae | Bathydemersal | Gulf hake | 32 | 188-636 |
| 251 | *Urophycis floridana* | Phycidae | Demersal | Southern codling | 7 | 192-423 |
| 252 | *Urophycis regia* | Phycidae | Demersal | Spotted codling | 5 | 183-538 |
| 121 | *Holtbyrnia innesi* | Platytroctidae | Bathypelagic | Teardrop tubeshoulder | 1 | 2243 |
| 192 | *Platytroctes apus* | Platytroctidae | Bathypelagic | Legless searsid | 1 | 3590 |
| 193 | *Poecilopsetta beanii* | Pleuronectidae | Bathydemersal | Deepwater dab | 27 | 192-1189 |
| 198 | *Polymixia lowei* | Polymixiidae | Bathydemersal | Beardfish | 9 | 229-636 |
| 73 | *Cruriraja cadenati* | Rajidae | Bathydemersal | Broadfoot leg skate | 1 | 345 |
| 74 | *Cruriraja rugosa* | Rajidae | Bathydemersal | Rough leg skate | 4 | 522-786 |
| 99 | *Fenestraja sinusmexicanus* | Rajidae | Demersal | Gulf of Mexico pygmy skate | 19 | 342-1784 |
| 142 | *Leucoraja garmani* | Rajidae | Reef | Freckled skate | 4 | 192-369 |
| 143 | *Leucoraja lentiginosa* | Rajidae | Demersal | Speckled skate | 1 | 216 |
| 179 | *Ophichthus cruentifer* | Rajidae | Demersal | Margined snake eel | 1 | 654 |
| 210 | *Rajella bigelowi* | Rajidae | Bathydemersal | Bigelow's ray | 2 | 751-900 |
| 211 | *Rajella fuliginea* | Rajidae | Demersal | Sooty skate | 5 | 758-1061 |
| 212 | *Rajella purpuriventralis* | Rajidae | Bathydemersal | Purplebelly skate | 4 | 803-1438 |
| 214 | *Rhinochimaera atlantica* | Rhinochimaeridae | Bathydemersal | Straightnose rabbitfish | 1 | 758 |
| 224 | *Scombrolabrax heterolepis* | Scombrolabracidae | Bathypelagic | Longfin escolar | 1 | 1369 |
| 199 | *Pontinus longispinis* | Scorpaenidae | Demersal | Longspine scorpionfish | 12 | 183-351 |
| 225 | *Scorpaena plumieri* | Scorpaenidae | Reef | Pacific spotted scorpionfish | 1 | 379 |
| 11 | *Apristurus laurussonii* | Scyliorhinidae | Bathydemersal | Iceland catshark | 2 | 1002-1170 |
| 12 | *Apristurus parvipinnis* | Scyliorhinidae | Bathydemersal | Smallfin catshark | 3 | 751-850 |
| 13 | *Apristurus profundorum* | Scyliorhinidae | Bathydemersal | Deep-water catshark | 7 | 538-1136 |
| 226 | *Scyliorhinus retifer* | Scyliorhinidae | Demersal | Chain catshark | 2 | 354-461 |
| 115 | *Helicolenus dactylopterus* | Sebastidae | Bathydemersal | Blackbelly rosefish | 1 | 367 |
| 246 | *Trachyscorpia cristulata* | Sebastidae | Bathydemersal | Spiny scorpionfish | 2 | 613-616 |
| 116 | *Hemanthias leptus* | Serranidae | Demersal | Longtail bass | 1 | 192 |
| 117 | *Hemanthias vivanus* | Serranidae | Benthopelagic | Red barbier | 10 | 329-412 |
| 227 | *Setarches guentheri* | Setarchidae | Benthopelagic | Channeled rockfish | 12 | 329-465 |
| 230 | *Squalus cubensis* | Squalidae | Demersal | Cuban dogfish | 1 | 229 |
| 232 | *Stephanoberyx monae* | Stephanoberycidae | Bathydemersal |  | 20 | 686-1510 |
| 15 | *Argyropelecus aculeatus* | Sternoptychidae | Bathypelagic | Lovely hatchetfish | 3 | 767-1369 |
| 16 | *Argyropelecus affinis* | Sternoptychidae | Bathypelagic | Pacific hatchet fish | 1 | 1880 |
| 17 | *Argyropelecus gigas* | Sternoptychidae | Bathypelagic | Hatchetfish | 2 | 758-2735 |
| 196 | *Polyipnus clarus* | Sternoptychidae | Bathydemersal | Slope hatchetfish | 1 | 400 |
| 233 | *Sternoptyx diaphana* | Sternoptychidae | Bathypelagic | Diaphanous hatchet fish | 5 | 1100-2243 |
| 234 | *Sternoptyx pseudobscura* | Sternoptychidae | Bathypelagic | Highlight hatchetfish | 4 | 758-2735 |
| 19 | *Aristostomias xenostoma* | Stomiidae | Pelagic |  | 1 | 3010 |
| 26 | *Bathophilus pawneei* | Stomiidae | Bathypelagic | Pawnee dragonfish | 3 | 566-998 |
| 46 | *Borostomias antarcticus* | Stomiidae | Bathydemersal | Snaggletooth | 1 | 632 |
| 51 | *Chauliodus danae* | Stomiidae | Bathypelagic | Dana viperfish | 1 | 758 |
| 52 | *Chauliodus sloani* | Stomiidae | Bathypelagic | Sloane's viperfish | 13 | 400-3075 |
| 151 | *Malacosteus niger* | Stomiidae | Bathypelagic | Stoplight loosejaw | 6 | 576-3287 |
| 154 | *Melanostomias biseriatus* | Stomiidae | Bathypelagic |  | 1 | 937 |
| 188 | *Photostomias guernei* | Stomiidae | Bathypelagic |  | 2 | 998-2250 |
| 235 | *Stomias affinis* | Stomiidae | Bathypelagic | Günther's boafish | 1 | 632 |
| 86 | *Dysommina rugosa* | Synaphobranchidae | Benthopelagic |  | 4 | 465-628 |
| 114 | *Haptenchelys texis* | Synaphobranchidae | Bathydemersal |  | 1 | 3267 |
| 119 | *Histiobranchus bathybius* | Synaphobranchidae | Benthopelagic | Deep-water arrowtooth eel | 1 | 937 |
| 134 | *Ilyophis brunneus* | Synaphobranchidae | Bathypelagic | Muddy arrowtooth eel | 20 | 747-3267 |
| 240 | *Synaphobranchus affinis* | Synaphobranchidae | Benthopelagic | Grey cutthroat | 1 | 2243 |
| 241 | *Synaphobranchus oregoni* | Synaphobranchidae | Bathydemersal |  | 58 | 479-1510 |
| 37 | *Bathysaurus mollis* | Synodontidae | Bathydemersal | Highfin lizardfish | 3 | 2915-3403 |
| 221 | *Saurida normani* | Synodontidae | Reef | Shortjaw lizardfish | 1 | 216 |
| 243 | *Thaumatichthys pagidostomus* | Thaumatichthyidae | Bathydemersal |  | 1 | 1880 |
| 122 | *Hoplostethus mediterraneus* | Trachichthyidae | Benthopelagic | Mediterranean slimehead | 3 | 412-465 |
| 123 | *Hoplostethus occidentalis* | Trachichthyidae | Bathypelagic | Western roughy | 13 | 325-475 |
| 120 | *Hollardia hollardi* | Triacanthodidae | Bathydemersal | Reticulate spikefish | 1 | 192 |
| 180 | *Parahollardia lineata* | Triacanthodidae | Demersal | Jambeau | 1 | 192 |
| 45 | *Benthodesmus tenuis* | Trichiuridae | Benthopelagic | Slender frostfish | 4 | 325-686 |
| 247 | *Trichiurus lepturus* | Trichiuridae | Benthopelagic | Largehead hairtail | 1 | 1369 |
| 42 | *Bellator militaris* | Triglidae | Demersal | Horned searobin | 1 | 229 |
| 204 | *Prionotus beanii* | Triglidae | Demersal | Bean's searobin | 1 | 192 |
| 205 | *Prionotus rubio* | Triglidae | Demersal | Blackwing searobin | 2 | 183-192 |
| 206 | *Prionotus stearnsi* | Triglidae | Demersal | Shortwing searobin | 2 | 183-329 |
| 109 | *Gnathagnus egregius* | Uranoscopidae | Demersal | Freckled stargazer | 13 | 192-538 |
| 98 | *Exechodontes daidaleus* | Zoarcidae | Bathydemersal |  | 3 | 479-834 |
| 147 | *Lycenchelys bullisi* | Zoarcidae | Bathydemersal |  | 5 | 625-1136 |
|  |  |  |  |  |  |  |
