# Supplementary material for: Long-Term Observations of Epibenthic Fish Zonation in the Deep Northern Gulf of Mexico
Source: PLoS One. 2012 Oct 3;7(10):e46707. doi: 10.1371/journal.pone.0046707 (PMC3463567; doi:10.1371/journal.pone.0046707)
Supplement: Table S3 — Occurrence and abundance of deep-sea epibenthic fishes during the Alaminos , NGoMCS, and DGoMB surveys in the northern Gulf of Mexico. “Trawl” denotes the unique sample ID shared between Table S1 and S3. “Code” denotes the unique species ID shared between Table S2 and S3. “N” denotes number of specimen recovered from each trawl sample. (DOC) [file pone.0046707.s003.doc]

Table S3. Occurrence and abundance of deep-sea epibenthic fishes during the *Alaminos*, NGoMCS, and DGoMB surveys in the northern Gulf of Mexico. “Trawl” denotes the unique sample ID shared between Table S1 and S3. “Code” denotes the unique species ID shared between Table S2 and S3. “N” denotes number of specimen recovered from each trawl sample.

| **Trawl** | **Code** | **N** | **Trawl** | **Code** | **N** | **Trawl** | **Code** | **N** | **Trawl** | **Code** | **N** | **Trawl** | **Code** | **N** |
| --- | --- | --- | --- | --- | --- | --- | --- | --- | --- | --- | --- | --- | --- | --- |
|  |  |  |  |  |  |  |  |  |  |  |  |  |  |  |
| 63 | 200 | NA | 199 | 81 | NA | 2E2 | 144 | 1 | 4E2A | 31 | 3 | 5WC9 | 29 | 13 |
| 63 | 253 | NA | 199 | 126 | NA | 2E2 | 170 | 7 | 4E2A | 53 | 40 | 5WC9 | 53 | 4 |
| 63 | 258 | NA | 199 | 155 | NA | 2E2 | 185 | 1 | 4E2A | 72 | 8 | 5WC9 | 62 | 2 |
| 64 | 30 | NA | 199 | 158 | NA | 2E2 | 193 | 2 | 4E2A | 81 | 59 | 5WC9 | 70 | 1 |
| 65 | 3 | NA | 199 | 193 | NA | 2E2 | 208 | 2 | 4E2A | 95 | 4 | 5WC9 | 72 | 3 |
| 66 | 3 | NA | 199 | 198 | NA | 2E3 | 29 | 4 | 4E2A | 98 | 1 | 5WC9 | 81 | 26 |
| 66 | 159 | NA | 199 | 199 | NA | 2E3 | 53 | 1 | 4E2A | 99 | 2 | 5WC9 | 95 | 1 |
| 66 | 241 | NA | 199 | 207 | NA | 2E3 | 70 | 2 | 4E2A | 146 | 2 | 5WC9 | 101 | 1 |
| 68 | 81 | NA | 199 | 208 | NA | 2E3 | 81 | 9 | 4E2A | 147 | 1 | 5WC9 | 103 | 1 |
| 68 | 95 | NA | 199 | 230 | NA | 2E3 | 103 | 1 | 4E2A | 155 | 2 | 5WC9 | 105 | 1 |
| 68 | 105 | NA | 199 | 231 | NA | 2E3 | 134 | 2 | 4E2A | 170 | 82 | 5WC9 | 112 | 3 |
| 68 | 130 | NA | 199 | 250 | NA | 2E3 | 141 | 1 | 4E2A | 171 | 3 | 5WC9 | 147 | 1 |
| 70 | 4 | NA | 199 | 257 | NA | 2E3 | 170 | 2 | 4E2A | 208 | 4 | 5WC9 | 170 | 6 |
| 70 | 70 | NA | 204 | 8 | NA | 2E3 | 173 | 1 | 4E2A | 236 | 2 | 5WC9 | 176 | 1 |
| 70 | 232 | NA | 204 | 43 | NA | 2E3 | 175 | 1 | 4E2A | 241 | 4 | 5WC9 | 208 | 4 |
| 72 | 28 | NA | 204 | 59 | NA | 2E3 | 241 | 24 | 4E2A | 250 | 3 | 5WC9 | 210 | 1 |
| 72 | 29 | NA | 204 | 79 | NA | 2E4 | 2 | 1 | 4E2B | 22 | 7 | 5WC9 | 241 | 39 |
| 72 | 53 | NA | 204 | 81 | NA | 2E4 | 3 | 4 | 4E2B | 53 | 12 | 1-B1-1 | 6 | 1 |
| 72 | 84 | NA | 204 | 100 | NA | 2E4 | 4 | 1 | 4E2B | 81 | 38 | 1-B1-1 | 83 | 1 |
| 72 | 95 | NA | 204 | 109 | NA | 2E4 | 11 | 1 | 4E2B | 95 | 2 | 1-B1-1 | 111 | 1 |
| 72 | 166 | NA | 204 | 116 | NA | 2E4 | 27 | 10 | 4E2B | 99 | 1 | 1-B1-1 | 188 | 1 |
| 72 | 170 | NA | 204 | 120 | NA | 2E4 | 28 | 2 | 4E2B | 138 | 7 | 1-B1-1 | 200 | 1 |
| 72 | 197 | NA | 204 | 124 | NA | 2E4 | 34 | 4 | 4E2B | 150 | 2 | 1-B2-3 | 5 | 1 |
| 72 | 208 | NA | 204 | 125 | NA | 2E4 | 35 | 9 | 4E2B | 170 | 11 | 1-B2-3 | 80 | 1 |
| 72 | 236 | NA | 204 | 126 | NA | 2E4 | 36 | 1 | 4E2B | 171 | 1 | 1-B2-3 | 135 | 3 |
| 79 | 44 | NA | 204 | 140 | NA | 2E4 | 43 | 1 | 4E2B | 208 | 2 | 1-B2-3 | 213 | 1 |
| 79 | 61 | NA | 204 | 142 | NA | 2E4 | 70 | 1 | 4E2B | 236 | 1 | 1-B2-3 | 233 | 1 |
| 79 | 81 | NA | 204 | 148 | NA | 2E4 | 103 | 1 | 4E2B | 241 | 4 | 1-B3-1 | 69 | 1 |
| 79 | 185 | NA | 204 | 156 | NA | 2E4 | 105 | 31 | 4E2B | 246 | 2 | 1-B3-1 | 83 | 1 |
| 79 | 225 | NA | 204 | 164 | NA | 2E4 | 112 | 1 | 4E2C | 13 | 1 | 1-C1-1 | 14 | 1 |
| 81 | 83 | NA | 204 | 180 | NA | 2E4 | 134 | 11 | 4E2C | 28 | 3 | 1-C1-1 | 44 | 3 |
| 81 | 135 | NA | 204 | 193 | NA | 2E4 | 173 | 10 | 4E2C | 29 | 1 | 1-C1-1 | 45 | 1 |
| 82 | 24 | NA | 204 | 199 | NA | 2E4 | 175 | 4 | 4E2C | 53 | 11 | 1-C1-1 | 49 | 3 |
| 82 | 30 | NA | 204 | 204 | NA | 2E4 | 182 | 4 | 4E2C | 74 | 1 | 1-C1-1 | 60 | 30 |
| 84 | 24 | NA | 204 | 205 | NA | 2E4 | 185 | 1 | 4E2C | 81 | 34 | 1-C1-1 | 77 | 3 |
| 94 | 81 | NA | 204 | 207 | NA | 2E4 | 217 | 1 | 4E2C | 99 | 1 | 1-C1-1 | 123 | 1 |
| 94 | 102 | NA | 204 | 248 | NA | 2E4 | 229 | 1 | 4E2C | 138 | 3 | 1-C1-1 | 182 | 1 |
| 94 | 112 | NA | 204 | 249 | NA | 2E4 | 232 | 33 | 4E2C | 168 | 1 | 1-C1-1 | 186 | 1 |
| 94 | 185 | NA | 204 | 250 | NA | 2E4 | 241 | 18 | 4E2C | 170 | 8 | 1-C1-1 | 193 | 2 |
| 94 | 241 | NA | 204 | 251 | NA | 2E4 | 245 | 1 | 4E2C | 171 | 2 | 1-C1-1 | 198 | 2 |
| 94 | 259 | NA | 204 | 252 | NA | 2E4 | 253 | 6 | 4E2C | 208 | 1 | 1-C1-1 | 199 | 18 |
| 97 | 81 | NA | 204 | 256 | NA | 2E4 | 258 | 2 | 4E2C | 241 | 9 | 1-C1-1 | 231 | 29 |
| 97 | 129 | NA | 206 | 28 | NA | 2E5 | 253 | 1 | 4E2D | 22 | 3 | 1-C1-1 | 238 | 3 |
| 99 | 1 | NA | 206 | 61 | NA | 2W1 | 44 | 45 | 4E2D | 28 | 5 | 1-C1-1 | 250 | 1 |
| 100 | 38 | NA | 206 | 81 | NA | 2W1 | 56 | 35 | 4E2D | 36 | 1 | 1-C1-1 | 261 | 2 |
| 102 | 81 | NA | 206 | 102 | NA | 2W1 | 60 | 3 | 4E2D | 53 | 18 | 1-C12-1 | 1 | 1 |
| 103 | 43 | NA | 206 | 133 | NA | 2W1 | 61 | 4 | 4E2D | 81 | 23 | 1-C12-1 | 21 | 1 |
| 104 | 140 | NA | 206 | 150 | NA | 2W1 | 90 | 15 | 4E2D | 86 | 1 | 1-C12-1 | 32 | 1 |
| 104 | 190 | NA | 206 | 170 | NA | 2W1 | 99 | 3 | 4E2D | 94 | 1 | 1-C12-1 | 37 | 4 |
| 104 | 193 | NA | 206 | 185 | NA | 2W1 | 101 | 5 | 4E2D | 95 | 3 | 1-C4-1 | 39 | 1 |
| 104 | 199 | NA | 206 | 252 | NA | 2W1 | 109 | 3 | 4E2D | 99 | 1 | 1-C4-1 | 52 | 2 |
| 105 | 44 | NA | 209 | 173 | NA | 2W1 | 117 | 2 | 4E2D | 113 | 1 | 1-C4-1 | 65 | 2 |
| 105 | 47 | NA | 210 | 3 | NA | 2W1 | 123 | 1 | 4E2D | 138 | 1 | 1-C4-1 | 70 | 4 |
| 105 | 56 | NA | 210 | 4 | NA | 2W1 | 133 | 3 | 4E2D | 155 | 2 | 1-C4-1 | 82 | 4 |
| 105 | 61 | NA | 210 | 27 | NA | 2W1 | 150 | 4 | 4E2D | 168 | 1 | 1-C4-1 | 105 | 7 |
| 105 | 81 | NA | 210 | 29 | NA | 2W1 | 155 | 3 | 4E2D | 170 | 12 | 1-C4-1 | 159 | 6 |
| 105 | 99 | NA | 210 | 70 | NA | 2W1 | 182 | 1 | 4E2D | 171 | 6 | 1-C4-1 | 173 | 7 |
| 105 | 150 | NA | 210 | 72 | NA | 2W1 | 185 | 2 | 4E2D | 208 | 10 | 1-C4-1 | 175 | 4 |
| 105 | 156 | NA | 210 | 81 | NA | 2W1 | 186 | 1 | 4E2D | 223 | 1 | 1-C4-1 | 183 | 2 |
| 105 | 185 | NA | 210 | 82 | NA | 2W1 | 193 | 24 | 4E2D | 238 | 1 | 1-C4-1 | 212 | 1 |
| 105 | 237 | NA | 210 | 103 | NA | 2W1 | 197 | 2 | 4E2D | 241 | 5 | 1-C4-1 | 229 | 1 |
| 106 | 26 | NA | 210 | 105 | NA | 2W1 | 199 | 1 | 4E2E | 28 | 3 | 1-C4-1 | 233 | 3 |
| 106 | 61 | NA | 210 | 112 | NA | 2W1 | 227 | 59 | 4E2E | 53 | 12 | 1-C4-1 | 241 | 5 |
| 106 | 99 | NA | 210 | 159 | NA | 2W1 | 239 | 1 | 4E2E | 81 | 1 | 1-C7-1 | 26 | 1 |
| 106 | 170 | NA | 210 | 170 | NA | 2W1 | 250 | 8 | 4E2E | 155 | 1 | 1-C7-1 | 52 | 2 |
| 106 | 189 | NA | 210 | 172 | NA | 2W2 | 28 | 3 | 4E2E | 170 | 3 | 1-C7-1 | 76 | 2 |
| 106 | 190 | NA | 210 | 173 | NA | 2W2 | 44 | 1 | 4E2E | 171 | 1 | 1-C7-1 | 110 | 1 |
| 106 | 236 | NA | 210 | 232 | NA | 2W2 | 53 | 2 | 4E2E | 176 | 1 | 1-C7-1 | 111 | 2 |
| 106 | 259 | NA | 210 | 241 | NA | 2W2 | 72 | 1 | 4E2E | 208 | 4 | 1-C7-1 | 151 | 1 |
| 107 | 29 | NA | 210 | 259 | NA | 2W2 | 81 | 15 | 4E2E | 222 | 1 | 1-C7-1 | 157 | 1 |
| 107 | 53 | NA | 213 | 4 | NA | 2W2 | 138 | 2 | 4E2E | 241 | 1 | 1-C7-1 | 188 | 1 |
| 107 | 70 | NA | 213 | 27 | NA | 2W2 | 155 | 1 | 4E3A | 13 | 1 | 1-MT1-1 | 28 | 26 |
| 107 | 72 | NA | 213 | 35 | NA | 2W2 | 168 | 1 | 4E3A | 29 | 36 | 1-MT1-1 | 44 | 6 |
| 107 | 81 | NA | 213 | 70 | NA | 2W2 | 170 | 5 | 4E3A | 70 | 6 | 1-MT1-1 | 60 | 18 |
| 107 | 82 | NA | 213 | 72 | NA | 2W2 | 179 | 1 | 4E3A | 72 | 2 | 1-MT1-1 | 61 | 33 |
| 109 | 29 | NA | 213 | 81 | NA | 2W2 | 259 | 1 | 4E3A | 81 | 10 | 1-MT1-1 | 81 | 6 |
| 109 | 72 | NA | 213 | 82 | NA | 2W3 | 29 | 2 | 4E3A | 103 | 2 | 1-MT1-1 | 90 | 3 |
| 109 | 82 | NA | 213 | 103 | NA | 2W3 | 36 | 1 | 4E3A | 105 | 11 | 1-MT1-1 | 95 | 3 |
| 109 | 105 | NA | 213 | 105 | NA | 2W3 | 70 | 5 | 4E3A | 112 | 5 | 1-MT1-1 | 101 | 2 |
| 109 | 112 | NA | 213 | 112 | NA | 2W3 | 81 | 2 | 4E3A | 134 | 3 | 1-MT1-1 | 123 | 1 |
| 109 | 159 | NA | 213 | 134 | NA | 2W3 | 88 | 2 | 4E3A | 155 | 1 | 1-MT1-1 | 139 | 33 |
| 109 | 170 | NA | 213 | 151 | NA | 2W3 | 90 | 3 | 4E3A | 170 | 15 | 1-MT1-1 | 150 | 1 |
| 109 | 210 | NA | 213 | 159 | NA | 2W3 | 112 | 7 | 4E3A | 171 | 1 | 1-MT1-1 | 155 | 2 |
| 109 | 241 | NA | 213 | 172 | NA | 2W3 | 173 | 2 | 4E3A | 173 | 9 | 1-MT1-1 | 173 | 1 |
| 109 | 253 | NA | 213 | 229 | NA | 2W3 | 175 | 2 | 4E3A | 241 | 23 | 1-MT1-1 | 226 | 1 |
| 110 | 4 | NA | 213 | 232 | NA | 2W3 | 241 | 27 | 4E3B | 28 | 1 | 1-MT1-1 | 227 | 4 |
| 110 | 35 | NA | 213 | 241 | NA | 2W4 | 3 | 2 | 4E3B | 29 | 9 | 1-MT1-1 | 231 | 4 |
| 110 | 65 | NA | 213 | 259 | NA | 2W4 | 135 | 1 | 4E3B | 31 | 1 | 1-MT1-1 | 250 | 21 |
| 110 | 82 | NA | 214 | 13 | NA | 2W4 | 232 | 1 | 4E3B | 70 | 3 | 1-MT1-1 | 259 | 10 |
| 110 | 105 | NA | 214 | 29 | NA | 2W4 | 241 | 1 | 4E3B | 81 | 2 | 2-MT1-1 | 52 | 2 |
| 110 | 134 | NA | 214 | 43 | NA | 3C1 | 44 | 4 | 4E3B | 98 | 1 | 2-MT1-1 | 109 | 1 |
| 110 | 159 | NA | 214 | 44 | NA | 3C1 | 56 | 5 | 4E3B | 134 | 3 | 2-MT1-1 | 187 | 1 |
| 110 | 170 | NA | 214 | 56 | NA | 3C1 | 60 | 20 | 4E3B | 173 | 3 | 2-MT1-1 | 236 | 2 |
| 110 | 229 | NA | 214 | 61 | NA | 3C1 | 90 | 2 | 4E3B | 211 | 4 | 1-MT2-1 | 28 | 13 |
| 110 | 232 | NA | 214 | 81 | NA | 3C1 | 99 | 1 | 4E3B | 241 | 7 | 1-MT2-1 | 45 | 1 |
| 110 | 241 | NA | 214 | 95 | NA | 3C1 | 101 | 10 | 4E3C | 13 | 1 | 1-MT2-1 | 72 | 19 |
| 111 | 3 | NA | 214 | 96 | NA | 3C1 | 109 | 1 | 4E3C | 29 | 12 | 1-MT2-1 | 81 | 1 |
| 111 | 4 | NA | 214 | 109 | NA | 3C1 | 117 | 1 | 4E3C | 70 | 3 | 1-MT2-1 | 82 | 3 |
| 111 | 35 | NA | 214 | 128 | NA | 3C1 | 123 | 1 | 4E3C | 72 | 1 | 1-MT2-1 | 84 | 1 |
| 111 | 65 | NA | 214 | 133 | NA | 3C1 | 142 | 1 | 4E3C | 81 | 12 | 1-MT2-1 | 103 | 1 |
| 111 | 82 | NA | 214 | 138 | NA | 3C1 | 150 | 5 | 4E3C | 105 | 8 | 1-MT2-1 | 141 | 1 |
| 112 | 70 | NA | 214 | 150 | NA | 3C1 | 152 | 3 | 4E3C | 112 | 1 | 1-MT2-1 | 170 | 49 |
| 112 | 82 | NA | 214 | 156 | NA | 3C1 | 155 | 2 | 4E3C | 134 | 4 | 1-MT2-1 | 232 | 1 |
| 112 | 105 | NA | 214 | 165 | NA | 3C1 | 182 | 2 | 4E3C | 170 | 3 | 1-MT3-1 | 11 | 2 |
| 112 | 228 | NA | 214 | 185 | NA | 3C1 | 193 | 4 | 4E3C | 173 | 2 | 1-MT3-1 | 29 | 3 |
| 112 | 232 | NA | 214 | 191 | NA | 3C1 | 198 | 3 | 4E3C | 195 | 2 | 1-MT3-1 | 48 | 1 |
| 112 | 241 | NA | 214 | 250 | NA | 3C1 | 231 | 27 | 4E3C | 241 | 22 | 1-MT3-1 | 70 | 6 |
| 113 | 4 | NA | 214 | 252 | NA | 3C1 | 238 | 5 | 4E3D | 13 | 1 | 1-MT3-1 | 72 | 16 |
| 113 | 65 | NA | 214 | 253 | NA | 3C1 | 250 | 20 | 4E3D | 29 | 3 | 1-MT3-1 | 82 | 10 |
| 113 | 82 | NA | 214 | 259 | NA | 3C2 | 28 | 6 | 4E3D | 72 | 1 | 1-MT3-1 | 105 | 2 |
| 113 | 200 | NA | 215 | 27 | NA | 3C2 | 29 | 3 | 4E3D | 81 | 7 | 1-MT3-1 | 111 | 1 |
| 114 | 4 | NA | 215 | 35 | NA | 3C2 | 46 | 1 | 4E3D | 101 | 1 | 1-MT3-1 | 113 | 1 |
| 114 | 24 | NA | 215 | 72 | NA | 3C2 | 53 | 1 | 4E3D | 103 | 1 | 1-MT3-1 | 130 | 2 |
| 114 | 200 | NA | 215 | 81 | NA | 3C2 | 61 | 7 | 4E3D | 105 | 1 | 1-MT3-1 | 159 | 3 |
| 115 | 4 | NA | 215 | 82 | NA | 3C2 | 72 | 3 | 4E3D | 134 | 3 | 1-MT3-1 | 173 | 61 |
| 115 | 67 | NA | 215 | 103 | NA | 3C2 | 81 | 18 | 4E3D | 147 | 1 | 1-MT3-1 | 203 | 1 |
| 115 | 72 | NA | 215 | 159 | NA | 3C2 | 92 | 1 | 4E3D | 153 | 1 | 1-MT3-1 | 229 | 14 |
| 115 | 82 | NA | 215 | 170 | NA | 3C2 | 93 | 1 | 4E3D | 162 | 1 | 1-MT4-1 | 15 | 1 |
| 115 | 105 | NA | 215 | 232 | NA | 3C2 | 99 | 2 | 4E3D | 168 | 1 | 1-MT4-1 | 65 | 1 |
| 115 | 134 | NA | 215 | 241 | NA | 3C2 | 137 | 1 | 4E3D | 170 | 2 | 1-MT4-1 | 70 | 3 |
| 115 | 159 | NA | 215 | 253 | NA | 3C2 | 138 | 6 | 4E3D | 173 | 4 | 1-MT4-1 | 81 | 1 |
| 115 | 170 | NA | 215 | 259 | NA | 3C2 | 150 | 2 | 4E3D | 241 | 21 | 1-MT4-1 | 82 | 9 |
| 115 | 229 | NA | 217 | 65 | NA | 3C2 | 168 | 2 | 5WC1 | 14 | 2 | 1-MT4-1 | 111 | 1 |
| 115 | 232 | NA | 218 | 140 | NA | 3C2 | 170 | 19 | 5WC1 | 43 | 1 | 1-MT4-1 | 128 | 1 |
| 115 | 241 | NA | 218 | 199 | NA | 3C2 | 235 | 1 | 5WC1 | 44 | 10 | 1-MT4-1 | 224 | 1 |
| 116 | 29 | NA | 218 | 248 | NA | 3C2 | 241 | 1 | 5WC1 | 56 | 27 | 1-MT4-1 | 232 | 4 |
| 116 | 72 | NA | 219 | 70 | NA | 3C2 | 250 | 7 | 5WC1 | 60 | 10 | 1-MT4-1 | 241 | 1 |
| 116 | 82 | NA | 222 | 35 | NA | 3C2 | 259 | 1 | 5WC1 | 61 | 1 | 1-MT4-1 | 247 | 1 |
| 116 | 105 | NA | 223 | 35 | NA | 3C3 | 12 | 1 | 5WC1 | 89 | 1 | 1-MT5-1 | 9 | 1 |
| 116 | 112 | NA | 223 | 253 | NA | 3C3 | 29 | 4 | 5WC1 | 90 | 12 | 1-MT5-1 | 25 | 2 |
| 116 | 134 | NA | 224 | 24 | NA | 3C3 | 70 | 6 | 5WC1 | 117 | 2 | 1-MT5-1 | 40 | 1 |
| 116 | 159 | NA | 224 | 33 | NA | 3C3 | 72 | 2 | 5WC1 | 123 | 2 | 1-MT5-1 | 69 | 2 |
| 116 | 170 | NA | 224 | 151 | NA | 3C3 | 81 | 15 | 5WC1 | 133 | 15 | 1-MT5-1 | 111 | 3 |
| 116 | 241 | NA | 225 | 22 | NA | 3C3 | 101 | 1 | 5WC1 | 142 | 1 | 1-MT5-1 | 121 | 1 |
| 117 | 200 | NA | 225 | 24 | NA | 3C3 | 112 | 2 | 5WC1 | 145 | 1 | 1-MT5-1 | 201 | 2 |
| 118 | 29 | NA | 225 | 114 | NA | 3C3 | 170 | 7 | 5WC1 | 150 | 4 | 1-MT5-1 | 233 | 1 |
| 118 | 70 | NA | 225 | 134 | NA | 3C3 | 212 | 2 | 5WC1 | 182 | 2 | 1-MT5-1 | 240 | 1 |
| 118 | 82 | NA | 257 | 3 | NA | 3C3 | 241 | 19 | 5WC1 | 185 | 3 | 1-MT6-1 | 17 | 1 |
| 118 | 151 | NA | 258 | 5 | NA | 3C3 | 259 | 5 | 5WC1 | 193 | 5 | 1-MT6-1 | 52 | 1 |
| 118 | 170 | NA | 258 | 13 | NA | 3C4 | 70 | 1 | 5WC1 | 198 | 2 | 1-MT6-1 | 71 | 1 |
| 118 | 241 | NA | 258 | 22 | NA | 3C4 | 232 | 1 | 5WC1 | 227 | 18 | 1-MT6-1 | 75 | 2 |
| 119 | 29 | NA | 258 | 29 | NA | 3C4 | 241 | 2 | 5WC1 | 231 | 1 | 1-MT6-1 | 111 | 2 |
| 119 | 70 | NA | 258 | 35 | NA | 3C5 | 1 | 2 | 5WC1 | 250 | 5 | 1-MT6-1 | 234 | 1 |
| 119 | 112 | NA | 258 | 38 | NA | 3C5 | 32 | 3 | 5WC10 | 28 | 1 | 1-NB2-1 | 258 | 1 |
| 119 | 159 | NA | 258 | 62 | NA | 3C5 | 71 | 2 | 5WC10 | 72 | 2 | 1-NB3-1 | 16 | 2 |
| 119 | 170 | NA | 258 | 70 | NA | 3C6 | 7 | 1 | 5WC10 | 74 | 1 | 1-NB3-1 | 25 | 1 |
| 119 | 241 | NA | 258 | 72 | NA | 3C6 | 44 | 12 | 5WC10 | 81 | 5 | 1-NB3-1 | 83 | 2 |
| 119 | 245 | NA | 258 | 81 | NA | 3C6 | 53 | 3 | 5WC10 | 113 | 1 | 1-NB3-1 | 111 | 2 |
| 121 | 81 | NA | 258 | 82 | NA | 3C6 | 61 | 46 | 5WC10 | 134 | 1 | 1-NB3-1 | 163 | 1 |
| 121 | 150 | NA | 258 | 105 | NA | 3C6 | 81 | 31 | 5WC10 | 168 | 1 | 1-NB3-1 | 177 | 1 |
| 121 | 155 | NA | 258 | 150 | NA | 3C6 | 90 | 7 | 5WC10 | 170 | 2 | 1-NB3-1 | 233 | 2 |
| 121 | 170 | NA | 258 | 159 | NA | 3C6 | 92 | 1 | 5WC10 | 241 | 3 | 1-NB3-1 | 243 | 1 |
| 121 | 241 | NA | 258 | 170 | NA | 3C6 | 93 | 15 | 5WC10 | 245 | 1 | 1-NB5-1 | 4 | 1 |
| 121 | 252 | NA | 258 | 211 | NA | 3C6 | 97 | 1 | 5WC11 | 4 | 11 | 1-NB5-1 | 25 | 1 |
| 122 | 43 | NA | 258 | 228 | NA | 3C6 | 101 | 1 | 5WC11 | 13 | 1 | 1-NB5-1 | 83 | 1 |
| 122 | 60 | NA | 258 | 232 | NA | 3C6 | 123 | 2 | 5WC11 | 27 | 2 | 1-NB5-1 | 135 | 1 |
| 122 | 81 | NA | 258 | 245 | NA | 3C6 | 138 | 16 | 5WC11 | 35 | 2 | 1-NB5-1 | 202 | 1 |
| 122 | 109 | NA | 258 | 253 | NA | 3C6 | 150 | 12 | 5WC11 | 41 | 1 | 1-NB5-1 | 218 | 1 |
| 122 | 150 | NA | 259 | 44 | NA | 3C6 | 155 | 1 | 5WC11 | 70 | 2 | 1-NB5-1 | 234 | 1 |
| 122 | 193 | NA | 259 | 56 | NA | 3C6 | 170 | 4 | 5WC11 | 99 | 1 | 1-NB5-1 | 253 | 1 |
| 122 | 231 | NA | 259 | 57 | NA | 3C6 | 185 | 13 | 5WC11 | 105 | 110 | 1-RW1-1 | 8 | 1 |
| 122 | 250 | NA | 259 | 60 | NA | 3C6 | 236 | 1 | 5WC11 | 112 | 6 | 1-RW1-1 | 43 | 2 |
| 123 | 43 | NA | 259 | 61 | NA | 3C6 | 250 | 100 | 5WC11 | 134 | 8 | 1-RW1-1 | 140 | 3 |
| 123 | 44 | NA | 259 | 102 | NA | 3C7 | 2 | 1 | 5WC11 | 135 | 9 | 1-RW1-1 | 199 | 1 |
| 123 | 60 | NA | 259 | 109 | NA | 3C7 | 29 | 1 | 5WC11 | 147 | 2 | 1-RW1-1 | 239 | 1 |
| 123 | 133 | NA | 259 | 117 | NA | 3C7 | 35 | 1 | 5WC11 | 195 | 1 | 1-RW1-1 | 248 | 1 |
| 123 | 150 | NA | 259 | 122 | NA | 3C7 | 36 | 1 | 5WC11 | 232 | 15 | 1-RW1-1 | 250 | 1 |
| 123 | 182 | NA | 259 | 133 | NA | 3C7 | 70 | 3 | 5WC11 | 241 | 25 | 3-S1-1 | 1 | 2 |
| 123 | 193 | NA | 259 | 150 | NA | 3C7 | 72 | 1 | 5WC11 | 253 | 1 | 3-S1-1 | 39 | 2 |
| 123 | 231 | NA | 259 | 156 | NA | 3C7 | 81 | 3 | 5WC12 | 4 | 3 | 3-S1-1 | 135 | 1 |
| 124 | 29 | NA | 259 | 193 | NA | 3C7 | 103 | 1 | 5WC12 | 27 | 2 | 3-S1-1 | 192 | 1 |
| 124 | 82 | NA | 259 | 227 | NA | 3C7 | 105 | 8 | 5WC12 | 70 | 6 | 3-S3-1 | 18 | 2 |
| 124 | 141 | NA | 259 | 250 | NA | 3C7 | 112 | 6 | 5WC12 | 105 | 4 | 3-S3-1 | 20 | 1 |
| 124 | 193 | NA | 260 | 26 | NA | 3C7 | 134 | 5 | 5WC12 | 134 | 4 | 3-S3-1 | 127 | 1 |
| 124 | 208 | NA | 260 | 28 | NA | 3C7 | 173 | 1 | 5WC12 | 153 | 1 | 3-S3-1 | 217 | 1 |
| 124 | 241 | NA | 260 | 53 | NA | 3C7 | 212 | 1 | 5WC12 | 228 | 3 | 1-S35-1 | 28 | 39 |
| 124 | 245 | NA | 260 | 61 | NA | 3C7 | 241 | 11 | 5WC12 | 229 | 3 | 1-S35-1 | 45 | 1 |
| 127 | 4 | NA | 260 | 64 | NA | 3C7 | 253 | 1 | 5WC12 | 241 | 3 | 1-S35-1 | 52 | 1 |
| 127 | 35 | NA | 260 | 72 | NA | 3C8 | 2 | 9 | 5WC12 | 253 | 5 | 1-S35-1 | 54 | 9 |
| 127 | 82 | NA | 260 | 81 | NA | 3C8 | 3 | 4 | 5WC2 | 28 | 3 | 1-S35-1 | 63 | 1 |
| 127 | 105 | NA | 260 | 84 | NA | 3C8 | 4 | 1 | 5WC2 | 44 | 5 | 1-S35-1 | 72 | 26 |
| 127 | 194 | NA | 260 | 96 | NA | 3C8 | 22 | 1 | 5WC2 | 61 | 5 | 1-S35-1 | 81 | 2 |
| 127 | 245 | NA | 260 | 99 | NA | 3C8 | 27 | 2 | 5WC2 | 81 | 1 | 1-S35-1 | 95 | 13 |
| 128 | 4 | NA | 260 | 105 | NA | 3C8 | 29 | 1 | 5WC2 | 90 | 2 | 1-S35-1 | 99 | 7 |
| 128 | 50 | NA | 260 | 112 | NA | 3C8 | 35 | 5 | 5WC2 | 95 | 17 | 1-S35-1 | 103 | 1 |
| 128 | 70 | NA | 260 | 156 | NA | 3C8 | 70 | 5 | 5WC2 | 101 | 2 | 1-S35-1 | 111 | 2 |
| 128 | 82 | NA | 260 | 170 | NA | 3C8 | 71 | 1 | 5WC2 | 133 | 25 | 1-S35-1 | 112 | 1 |
| 128 | 105 | NA | 260 | 171 | NA | 3C8 | 105 | 8 | 5WC2 | 138 | 2 | 1-S35-1 | 128 | 4 |
| 128 | 151 | NA | 260 | 193 | NA | 3C8 | 134 | 37 | 5WC2 | 150 | 3 | 1-S35-1 | 155 | 10 |
| 128 | 159 | NA | 260 | 250 | NA | 3C8 | 173 | 6 | 5WC2 | 155 | 2 | 1-S35-1 | 167 | 2 |
| 128 | 193 | NA | 260 | 259 | NA | 3C8 | 175 | 1 | 5WC2 | 185 | 11 | 1-S35-1 | 170 | 25 |
| 128 | 232 | NA | 261 | 3 | NA | 3C8 | 195 | 2 | 5WC2 | 238 | 1 | 1-S35-1 | 259 | 8 |
| 128 | 241 | NA | 261 | 4 | NA | 3C8 | 228 | 6 | 5WC2 | 250 | 7 | 1-S36-1 | 3 | 3 |
| 128 | 253 | NA | 261 | 33 | NA | 3C8 | 232 | 3 | 5WC3 | 29 | 2 | 1-S36-1 | 4 | 1 |
| 130 | 241 | NA | 261 | 50 | NA | 3C8 | 241 | 23 | 5WC3 | 53 | 1 | 1-S36-1 | 6 | 1 |
| 132 | 161 | NA | 261 | 70 | NA | 3C8 | 253 | 1 | 5WC3 | 72 | 1 | 1-S36-1 | 25 | 1 |
| 132 | 199 | NA | 261 | 81 | NA | 3C10 | 3 | 3 | 5WC3 | 81 | 4 | 1-S36-1 | 52 | 1 |
| 134 | 44 | NA | 261 | 82 | NA | 3C10 | 4 | 1 | 5WC3 | 92 | 1 | 1-S36-1 | 65 | 1 |
| 134 | 60 | NA | 261 | 232 | NA | 3C10 | 22 | 1 | 5WC3 | 95 | 2 | 1-S36-1 | 69 | 2 |
| 134 | 109 | NA | 261 | 241 | NA | 3C10 | 35 | 1 | 5WC3 | 105 | 3 | 1-S36-1 | 81 | 2 |
| 134 | 193 | NA | 261 | 253 | NA | 3C11 | 32 | 2 | 5WC3 | 112 | 1 | 1-S36-1 | 82 | 4 |
| 134 | 219 | NA | 262 | 3 | NA | 3C11 | 70 | 1 | 5WC3 | 134 | 2 | 1-S36-1 | 83 | 4 |
| 135 | 22 | NA | 262 | 35 | NA | 3C11 | 71 | 1 | 5WC3 | 146 | 1 | 1-S36-1 | 85 | 1 |
| 135 | 81 | NA | 262 | 70 | NA | 4E1 | 14 | 2 | 5WC3 | 147 | 1 | 1-S36-1 | 99 | 1 |
| 135 | 84 | NA | 262 | 82 | NA | 4E1 | 43 | 1 | 5WC3 | 168 | 2 | 1-S36-1 | 111 | 1 |
| 135 | 133 | NA | 262 | 105 | NA | 4E1 | 44 | 40 | 5WC3 | 170 | 3 | 1-S36-1 | 146 | 2 |
| 135 | 150 | NA | 262 | 159 | NA | 4E1 | 56 | 106 | 5WC3 | 208 | 7 | 1-S36-1 | 163 | 1 |
| 135 | 151 | NA | 262 | 170 | NA | 4E1 | 61 | 24 | 5WC3 | 211 | 2 | 1-S36-1 | 234 | 1 |
| 135 | 171 | NA | 262 | 172 | NA | 4E1 | 81 | 1 | 5WC3 | 241 | 31 | 1-S36-1 | 258 | 1 |
| 137 | 33 | NA | 262 | 228 | NA | 4E1 | 90 | 30 | 5WC4 | 44 | 4 | 1-S37-1 | 4 | 2 |
| 137 | 170 | NA | 262 | 232 | NA | 4E1 | 99 | 1 | 5WC4 | 53 | 5 | 1-S37-1 | 6 | 1 |
| 137 | 241 | NA | 262 | 253 | NA | 4E1 | 109 | 1 | 5WC4 | 61 | 4 | 1-S37-1 | 71 | 1 |
| 137 | 259 | NA | 264 | 28 | NA | 4E1 | 117 | 3 | 5WC4 | 74 | 1 | 1-S37-1 | 83 | 3 |
| 138 | 81 | NA | 264 | 35 | NA | 4E1 | 118 | 1 | 5WC4 | 81 | 10 | 1-S37-1 | 111 | 1 |
| 138 | 82 | NA | 264 | 41 | NA | 4E1 | 133 | 9 | 5WC4 | 86 | 1 | 1-S37-1 | 163 | 1 |
| 138 | 112 | NA | 264 | 62 | NA | 4E1 | 145 | 1 | 5WC4 | 87 | 1 | 1-S37-1 | 200 | 2 |
| 139 | 24 | NA | 264 | 72 | NA | 4E1 | 150 | 4 | 5WC4 | 90 | 1 | 1-S38-1 | 1 | 2 |
| 139 | 65 | NA | 264 | 81 | NA | 4E1 | 155 | 41 | 5WC4 | 92 | 1 | 1-S38-1 | 25 | 2 |
| 139 | 82 | NA | 264 | 103 | NA | 4E1 | 182 | 12 | 5WC4 | 95 | 1 | 1-S38-1 | 32 | 1 |
| 139 | 232 | NA | 264 | 166 | NA | 4E1 | 185 | 25 | 5WC4 | 133 | 11 | 1-S38-1 | 69 | 3 |
| 139 | 241 | NA | 264 | 170 | NA | 4E1 | 193 | 33 | 5WC4 | 138 | 4 | 1-S38-1 | 108 | 1 |
| 139 | 253 | NA | 264 | 241 | NA | 4E1 | 198 | 3 | 5WC4 | 150 | 5 | 1-S38-1 | 163 | 2 |
| 140 | 105 | NA | 264 | 259 | NA | 4E1 | 220 | 1 | 5WC4 | 155 | 1 | 3-S4-1 | 37 | 1 |
| 140 | 134 | NA | 1C1 | 14 | 4 | 4E1 | 226 | 2 | 5WC4 | 170 | 1 | 1-S40-1 | 19 | 1 |
| 140 | 253 | NA | 1C1 | 56 | 5 | 4E1 | 227 | 2 | 5WC4 | 185 | 4 | 1-S40-1 | 23 | 1 |
| 142 | 35 | NA | 1C1 | 60 | 77 | 4E1 | 231 | 4 | 5WC4 | 236 | 2 | 1-S40-1 | 25 | 2 |
| 142 | 38 | NA | 1C1 | 61 | 3 | 4E1 | 238 | 3 | 5WC4 | 250 | 14 | 1-S40-1 | 34 | 1 |
| 142 | 65 | NA | 1C1 | 90 | 3 | 4E1 | 239 | 9 | 5WC4 | 255 | 1 | 1-S40-1 | 111 | 1 |
| 142 | 72 | NA | 1C1 | 109 | 1 | 4E1 | 250 | 16 | 5WC5 | 7 | 1 | 1-S41-1 | 1 | 3 |
| 142 | 82 | NA | 1C1 | 117 | 4 | 4E1 | 251 | 5 | 5WC5 | 44 | 16 | 1-S41-1 | 30 | 4 |
| 142 | 170 | NA | 1C1 | 123 | 1 | 4E2 | 22 | 2 | 5WC5 | 53 | 1 | 1-S41-1 | 52 | 1 |
| 142 | 232 | NA | 1C1 | 140 | 1 | 4E2 | 28 | 1 | 5WC5 | 56 | 39 | 1-S41-1 | 69 | 1 |
| 142 | 241 | NA | 1C1 | 142 | 1 | 4E2 | 53 | 9 | 5WC5 | 60 | 12 | 1-S41-1 | 111 | 1 |
| 142 | 253 | NA | 1C1 | 148 | 1 | 4E2 | 81 | 26 | 5WC5 | 81 | 16 | 1-S41-1 | 131 | 1 |
| 143 | 3 | NA | 1C1 | 150 | 5 | 4E2 | 95 | 2 | 5WC5 | 90 | 27 | 1-S41-1 | 201 | 1 |
| 143 | 82 | NA | 1C1 | 155 | 2 | 4E2 | 101 | 1 | 5WC5 | 99 | 2 | 1-S42-1 | 28 | 3 |
| 143 | 228 | NA | 1C1 | 182 | 16 | 4E2 | 138 | 4 | 5WC5 | 101 | 2 | 1-S42-1 | 63 | 1 |
| 145 | 4 | NA | 1C1 | 185 | 2 | 4E2 | 155 | 1 | 5WC5 | 109 | 5 | 1-S42-1 | 69 | 3 |
| 145 | 70 | NA | 1C1 | 186 | 3 | 4E2 | 170 | 7 | 5WC5 | 122 | 1 | 1-S42-1 | 72 | 1 |
| 145 | 82 | NA | 1C1 | 193 | 31 | 4E2 | 173 | 1 | 5WC5 | 123 | 4 | 1-S42-1 | 81 | 5 |
| 179 | 38 | NA | 1C1 | 198 | 2 | 4E2 | 241 | 3 | 5WC5 | 133 | 10 | 1-S42-1 | 82 | 7 |
| 184 | 44 | NA | 1C1 | 199 | 4 | 4E2 | 246 | 1 | 5WC5 | 138 | 15 | 1-S42-1 | 106 | 2 |
| 184 | 61 | NA | 1C1 | 206 | 1 | 4E2 | 250 | 1 | 5WC5 | 145 | 1 | 1-S42-1 | 111 | 6 |
| 184 | 81 | NA | 1C1 | 227 | 1 | 4E3 | 29 | 2 | 5WC5 | 150 | 20 | 1-S42-1 | 113 | 1 |
| 184 | 100 | NA | 1C1 | 231 | 2 | 4E3 | 70 | 1 | 5WC5 | 185 | 4 | 1-S42-1 | 155 | 1 |
| 184 | 174 | NA | 1C1 | 250 | 26 | 4E3 | 81 | 4 | 5WC5 | 186 | 1 | 1-S42-1 | 159 | 3 |
| 184 | 189 | NA | 1C1 | 251 | 5 | 4E3 | 101 | 1 | 5WC5 | 193 | 8 | 1-S42-1 | 166 | 3 |
| 184 | 236 | NA | 1C2 | 28 | 2 | 4E3 | 104 | 2 | 5WC5 | 198 | 3 | 1-S42-1 | 170 | 8 |
| 184 | 237 | NA | 1C2 | 53 | 3 | 4E3 | 112 | 2 | 5WC5 | 227 | 2 | 1-S42-1 | 241 | 1 |
| 185 | 44 | NA | 1C2 | 60 | 1 | 4E3 | 134 | 1 | 5WC5 | 231 | 1 | 1-S42-1 | 244 | 1 |
| 185 | 57 | NA | 1C2 | 72 | 1 | 4E3 | 141 | 1 | 5WC5 | 250 | 25 | 1-S42-1 | 259 | 41 |
| 185 | 193 | NA | 1C2 | 74 | 2 | 4E3 | 170 | 10 | 5WC5 | 251 | 1 | 2-S42-1 | 15 | 1 |
| 185 | 250 | NA | 1C2 | 78 | 1 | 4E3 | 173 | 2 | 5WC6 | 28 | 3 | 2-S42-1 | 52 | 2 |
| 186 | 43 | NA | 1C2 | 170 | 6 | 4E3 | 208 | 2 | 5WC6 | 53 | 8 | 1-S43-1 | 44 | 8 |
| 186 | 199 | NA | 1C2 | 208 | 4 | 4E3 | 241 | 3 | 5WC6 | 61 | 2 | 1-S43-1 | 60 | 5 |
| 186 | 205 | NA | 1C2 | 241 | 3 | 4ElA | 7 | 1 | 5WC6 | 81 | 33 | 1-S43-1 | 145 | 1 |
| 186 | 206 | NA | 1C3 | 12 | 1 | 4ElA | 14 | 1 | 5WC6 | 89 | 4 | 1-S43-1 | 155 | 1 |
| 186 | 215 | NA | 1C3 | 41 | 1 | 4ElA | 43 | 2 | 5WC6 | 95 | 9 | 1-S43-1 | 185 | 6 |
| 186 | 231 | NA | 1C3 | 70 | 4 | 4ElA | 44 | 68 | 5WC6 | 99 | 2 | 1-S43-1 | 193 | 1 |
| 186 | 252 | NA | 1C3 | 89 | 1 | 4ElA | 56 | 28 | 5WC6 | 101 | 1 | 1-S43-1 | 231 | 5 |
| 187 | 161 | NA | 1C3 | 105 | 6 | 4ElA | 60 | 20 | 5WC6 | 104 | 2 | 1-S43-1 | 238 | 1 |
| 187 | 169 | NA | 1C3 | 170 | 1 | 4ElA | 61 | 6 | 5WC6 | 138 | 6 | 1-S43-1 | 250 | 1 |
| 188 | 29 | NA | 1C3 | 241 | 6 | 4ElA | 81 | 4 | 5WC6 | 146 | 1 | 1-S43-1 | 254 | 3 |
| 188 | 72 | NA | 1C3 | 259 | 1 | 4ElA | 90 | 21 | 5WC6 | 150 | 1 | 1-S44-1 | 8 | 1 |
| 188 | 81 | NA | 2C1 | 14 | 1 | 4ElA | 117 | 8 | 5WC6 | 155 | 5 | 1-S44-1 | 10 | 34 |
| 188 | 82 | NA | 2C1 | 44 | 5 | 4ElA | 123 | 3 | 5WC6 | 168 | 2 | 1-S44-1 | 43 | 1 |
| 188 | 105 | NA | 2C1 | 56 | 1 | 4ElA | 133 | 4 | 5WC6 | 170 | 11 | 1-S44-1 | 58 | 1 |
| 188 | 112 | NA | 2C1 | 60 | 34 | 4ElA | 150 | 3 | 5WC6 | 171 | 1 | 1-S44-1 | 143 | 4 |
| 188 | 159 | NA | 2C1 | 61 | 7 | 4ElA | 155 | 11 | 5WC6 | 176 | 1 | 1-S44-1 | 158 | 2 |
| 188 | 168 | NA | 2C1 | 90 | 5 | 4ElA | 182 | 4 | 5WC6 | 185 | 2 | 1-S44-1 | 181 | 1 |
| 188 | 170 | NA | 2C1 | 117 | 1 | 4ElA | 185 | 5 | 5WC6 | 236 | 1 | 1-S44-1 | 186 | 10 |
| 188 | 241 | NA | 2C1 | 150 | 13 | 4ElA | 186 | 1 | 5WC6 | 238 | 1 | 1-S44-1 | 207 | 1 |
| 193 | 173 | NA | 2C1 | 155 | 3 | 4ElA | 193 | 15 | 5WC6 | 241 | 3 | 1-S44-1 | 221 | 2 |
| 194 | 29 | NA | 2C1 | 182 | 2 | 4ElA | 199 | 24 | 5WC7 | 44 | 16 | 1-S44-1 | 260 | 2 |
| 194 | 55 | NA | 2C1 | 193 | 13 | 4ElA | 216 | 1 | 5WC7 | 53 | 2 | 3-S5-1 | 37 | 2 |
| 194 | 70 | NA | 2C1 | 197 | 1 | 4ElA | 227 | 3 | 5WC7 | 56 | 26 | 3-S5-1 | 135 | 1 |
| 194 | 81 | NA | 2C1 | 199 | 1 | 4ElA | 231 | 2 | 5WC7 | 61 | 9 | 3-S5-1 | 160 | 1 |
| 194 | 82 | NA | 2C1 | 250 | 10 | 4ElA | 236 | 1 | 5WC7 | 81 | 76 | 1-W1-1 | 44 | 15 |
| 194 | 105 | NA | 2C1 | 251 | 1 | 4ElA | 238 | 5 | 5WC7 | 86 | 1 | 1-W1-1 | 45 | 2 |
| 194 | 112 | NA | 2C2 | 22 | 1 | 4ElA | 239 | 2 | 5WC7 | 87 | 1 | 1-W1-1 | 52 | 1 |
| 194 | 134 | NA | 2C2 | 28 | 2 | 4ElA | 250 | 11 | 5WC7 | 90 | 20 | 1-W1-1 | 56 | 27 |
| 194 | 159 | NA | 2C2 | 29 | 1 | 4E1B | 14 | 1 | 5WC7 | 91 | 3 | 1-W1-1 | 61 | 1 |
| 194 | 170 | NA | 2C2 | 53 | 3 | 4E1B | 43 | 2 | 5WC7 | 95 | 2 | 1-W1-1 | 77 | 1 |
| 194 | 241 | NA | 2C2 | 72 | 1 | 4E1B | 44 | 11 | 5WC7 | 97 | 8 | 1-W1-1 | 81 | 16 |
| 195 | 28 | NA | 2C2 | 81 | 1 | 4E1B | 56 | 8 | 5WC7 | 99 | 2 | 1-W1-1 | 90 | 5 |
| 195 | 29 | NA | 2C2 | 95 | 2 | 4E1B | 61 | 14 | 5WC7 | 101 | 4 | 1-W1-1 | 95 | 4 |
| 195 | 52 | NA | 2C2 | 113 | 1 | 4E1B | 73 | 4 | 5WC7 | 122 | 1 | 1-W1-1 | 99 | 2 |
| 195 | 72 | NA | 2C2 | 170 | 5 | 4E1B | 90 | 4 | 5WC7 | 123 | 1 | 1-W1-1 | 123 | 3 |
| 195 | 81 | NA | 2C2 | 193 | 1 | 4E1B | 109 | 1 | 5WC7 | 133 | 77 | 1-W1-1 | 132 | 15 |
| 195 | 82 | NA | 2C2 | 208 | 1 | 4E1B | 117 | 2 | 5WC7 | 138 | 21 | 1-W1-1 | 149 | 2 |
| 195 | 112 | NA | 2C2 | 259 | 1 | 4E1B | 150 | 11 | 5WC7 | 145 | 2 | 1-W1-1 | 155 | 3 |
| 195 | 119 | NA | 2C3 | 22 | 1 | 4E1B | 155 | 7 | 5WC7 | 150 | 6 | 1-W1-1 | 182 | 9 |
| 195 | 154 | NA | 2C3 | 28 | 1 | 4E1B | 182 | 2 | 5WC7 | 155 | 4 | 1-W1-1 | 193 | 1 |
| 195 | 159 | NA | 2C3 | 29 | 3 | 4E1B | 184 | 1 | 5WC7 | 182 | 4 | 1-W1-1 | 196 | 8 |
| 195 | 170 | NA | 2C3 | 72 | 1 | 4E1B | 185 | 7 | 5WC7 | 185 | 5 | 1-W1-1 | 197 | 1 |
| 195 | 178 | NA | 2C3 | 81 | 5 | 4E1B | 193 | 3 | 5WC7 | 227 | 1 | 1-W1-1 | 198 | 1 |
| 195 | 183 | NA | 2C3 | 170 | 2 | 4E1B | 227 | 1 | 5WC7 | 236 | 2 | 1-W1-1 | 250 | 2 |
| 195 | 211 | NA | 2C3 | 193 | 1 | 4E1B | 238 | 1 | 5WC7 | 238 | 1 | 1-W3-1 | 15 | 1 |
| 195 | 228 | NA | 2C3 | 241 | 4 | 4E1B | 239 | 1 | 5WC7 | 250 | 15 | 1-W3-1 | 66 | 1 |
| 195 | 241 | NA | 2C3 | 259 | 1 | 4E1B | 250 | 22 | 5WC8 | 44 | 31 | 1-W3-1 | 103 | 1 |
| 195 | 259 | NA | 2C4 | 170 | 1 | 4E1B | 251 | 1 | 5WC8 | 53 | 6 | 1-W3-1 | 173 | 1 |
| 196 | 28 | NA | 2C4 | 212 | 1 | 4E1B | 261 | 1 | 5WC8 | 56 | 1 | 1-W6-1 | 1 | 3 |
| 196 | 29 | NA | 2C5 | 253 | 1 | 4E1C | 44 | 69 | 5WC8 | 61 | 39 | 1-W6-1 | 21 | 1 |
| 196 | 53 | NA | 2E1 | 44 | 19 | 4E1C | 53 | 1 | 5WC8 | 81 | 63 | 1-W6-1 | 25 | 1 |
| 196 | 55 | NA | 2E1 | 56 | 10 | 4E1C | 56 | 13 | 5WC8 | 86 | 2 | 1-W6-1 | 32 | 1 |
| 196 | 68 | NA | 2E1 | 61 | 3 | 4E1C | 61 | 31 | 5WC8 | 90 | 12 | 1-W6-1 | 38 | 1 |
| 196 | 72 | NA | 2E1 | 90 | 6 | 4E1C | 90 | 5 | 5WC8 | 92 | 2 | 1-W6-1 | 52 | 1 |
| 196 | 81 | NA | 2E1 | 115 | 1 | 4E1C | 99 | 2 | 5WC8 | 95 | 15 | 1-WC12-1 | 52 | 1 |
| 196 | 84 | NA | 2E1 | 123 | 1 | 4E1C | 109 | 1 | 5WC8 | 97 | 3 | 1-WC12-1 | 209 | 1 |
| 196 | 94 | NA | 2E1 | 133 | 27 | 4E1C | 117 | 2 | 5WC8 | 98 | 2 | 1-WC12-1 | 233 | 1 |
| 196 | 101 | NA | 2E1 | 144 | 4 | 4E1C | 123 | 1 | 5WC8 | 101 | 3 | 1-WC5-1 | 17 | 1 |
| 196 | 102 | NA | 2E1 | 150 | 1 | 4E1C | 145 | 2 | 5WC8 | 133 | 71 | 1-WC5-1 | 51 | 1 |
| 196 | 112 | NA | 2E1 | 155 | 2 | 4E1C | 150 | 5 | 5WC8 | 138 | 26 | 1-WC5-1 | 52 | 1 |
| 196 | 156 | NA | 2E1 | 185 | 14 | 4E1C | 155 | 6 | 5WC8 | 145 | 1 | 1-WC5-1 | 62 | 1 |
| 196 | 161 | NA | 2E1 | 227 | 5 | 4E1C | 182 | 1 | 5WC8 | 150 | 5 | 1-WC5-1 | 82 | 5 |
| 196 | 171 | NA | 2E1 | 236 | 1 | 4E1C | 185 | 12 | 5WC8 | 155 | 3 | 1-WC5-1 | 105 | 5 |
| 196 | 198 | NA | 2E1 | 238 | 2 | 4E1C | 193 | 15 | 5WC8 | 170 | 1 | 1-WC5-1 | 107 | 1 |
| 196 | 241 | NA | 2E1 | 250 | 13 | 4E1C | 227 | 2 | 5WC8 | 182 | 2 | 1-WC5-1 | 111 | 2 |
| 196 | 250 | NA | 2E2 | 22 | 1 | 4E1C | 231 | 1 | 5WC8 | 185 | 17 | 1-WC5-1 | 136 | 1 |
| 196 | 259 | NA | 2E2 | 28 | 7 | 4E1C | 238 | 6 | 5WC8 | 236 | 1 | 1-WC5-1 | 173 | 1 |
| 199 | 8 | NA | 2E2 | 53 | 7 | 4E1C | 239 | 1 | 5WC8 | 241 | 1 | 1-WC5-1 | 211 | 1 |
| 199 | 14 | NA | 2E2 | 72 | 1 | 4E1C | 250 | 19 | 5WC8 | 250 | 52 | 1-WC5-1 | 214 | 1 |
| 199 | 42 | NA | 2E2 | 81 | 13 | 4E1C | 251 | 4 | 5WC9 | 12 | 1 | 1-WC5-1 | 234 | 1 |
| 199 | 43 | NA | 2E2 | 95 | 2 | 4E2A | 22 | 4 | 5WC9 | 22 | 2 | 1-WC5-1 | 242 | 2 |
| 199 | 61 | NA | 2E2 | 138 | 3 | 4E2A | 28 | 51 | 5WC9 | 28 | 12 | 1-WC5-1 | 259 | 1 |
|  |  |  |  |  |  |  |  |  |  |  |  |  |  |  |
