# Supplementary material for: Long-Term Observations of Epibenthic Fish Zonation in the Deep Northern Gulf of Mexico
Source: PLoS One. 2012 Oct 3;7(10):e46707. doi: 10.1371/journal.pone.0046707 (PMC3463567; doi:10.1371/journal.pone.0046707)
Supplement: Table S4 — The characteristic epibenthic fish species in the northern Gulf of Mexico. The top-10 species with the highest occurrence were listed for each faunal group based on the cluster analysis of pooled data. “Code” corresponds to the species code in Table S2. “Occur” denotes number of occurrence and “% Occur” denotes percentage of occurrence in specific faunal zones. (DOC) [file pone.0046707.s004.doc]

Table S4. The characteristic epibenthic fish species in the northern Gulf of Mexico. The top-10 species with the highest occurrence were listed for each faunal group based on the cluster analysis of pooled data. “Code” corresponds to the species code in Table S2. “Occur” denotes number of occurrence and “% Occur” denotes percentage of occurrence in specific faunal zones.

| Group | Code | Species Name | Family | Common Name | Occurrence | Trophic Level |
| --- | --- | --- | --- | --- | --- | --- |
|  |  |  |  |  |  |  |
| SB | 8 | *Ancylopsetta dilecta* | Paralichthyidae | Three-eye flounder | 4 | 3.78 |
|  | 43 | *Bembrops anatirostris* | Percophidae | Duckbill flathead | 14 | 4.26 |
|  | 161 | *Myrophis punctatus* | Ophichthidae | Speckled worm-eel | 3 | 3.85 |
|  | 199 | *Pontinus longispinis* | Scorpaenidae | Longspine scorpionfish | 12 | 3.49 |
|  | 10 | *Antigonia capros* | Caproidae | Deepbody boarfish | 1 | 4.05 |
|  | 58 | *Citharichthys cornutus* | Paralichthyidae | Horned whiff | 1 | 3.35 |
|  | 140 | *Lepophidium brevibarbe* | Ophidiidae | Shortbeard cusk-eel | 5 | 3.58 |
|  | 143 | *Leucoraja lentiginosa* | Rajidae | Speckled skate | 1 | 3.52 |
|  | 158 | *Monolene sessilicauda* | Bothidae | Deepwater flounder | 2 | 3.56 |
|  | 169 | *Nettenchelys pygmaea* | Nettastomatidae | Pygmy pikeconger | 1 | 3.29 |
|  |  |  |  |  |  |  |
| US | 250 | *Urophycis cirrata* | Phycidae | Gulf hake | 32 | 3.96 |
|  | 44 | *Bembrops gobioides* | Percophidae | Goby flathead | 28 | 4.19 |
|  | 150 | *Malacocephalus occidentalis* | Macrouridae | Western softhead grenadier | 29 | 3.69 |
|  | 193 | *Poecilopsetta beanii* | Pleuronectidae | Deepwater dab | 27 | 3.44 |
|  | 61 | *Coelorinchus coelorhincus* | Macrouridae | Hollowsnout grenadier | 27 | 3.6 |
|  | 90 | *Epigonus pandionis* | Epigonidae | Bigeye | 19 | 3.41 |
|  | 155 | *Merluccius albidus* | Merlucciidae | Offshore silver hake | 28 | 3.43 |
|  | 56 | *Chlorophthalmus agassizi* | Chlorophthalmidae | Shortnose greeneye | 17 | 3.66 |
|  | 185 | *Peristedion greyae* | Peristediidae |  | 23 | 3.47 |
|  | 60 | *Coelorinchus caribbaeus* | Macrouridae | Blackfin grenadier | 15 | 3.59 |
|  |  |  |  |  |  |  |
| U-MS | 241 | *Synaphobranchus oregoni* | Synaphobranchidae |  | 58 | 4.11 |
|  | 81 | *Dibranchus atlanticus* | Ogcocephalidae | Atlantic batfish | 66 | 3.42 |
|  | 170 | *Nezumia aequalis* | Macrouridae | Common Atlantic grenadier | 51 | 3.3 |
|  | 72 | *Coryphaenoides zaniophorus* | Macrouridae | Thickbeard grenadier | 33 | 3.23 |
|  | 70 | *Coryphaenoides mexicanus* | Macrouridae | Mexican grenadier | 33 | 3.53 |
|  | 82 | *Dicrolene introniger* | Ophidiidae | Digitate cusk eel | 33 | 3.03 |
|  | 29 | *Bathygadus melanobranchus* | Macrouridae | Vaillant's grenadier | 31 | 3.3 |
|  | 105 | *Gadomus longifilis* | Macrouridae | Treadfin grenadier | 30 | 3.17 |
|  | 28 | *Bathygadus macrops* | Macrouridae | Bullseye grenadier | 27 | 3.2 |
|  | 112 | *Halosaurus guentheri* | Halosauridae |  | 23 | 3.39 |
|  |  |  |  |  |  |  |
| M-LS+LS | 111 | *Gonostoma elongatum* | Gonostomatidae | Elongated bristlemouth fish | 14 | 3.3 |
|  | 52 | *Chauliodus sloani* | Stomiidae | Sloane's viperfish | 13 | 4.2 |
|  | 83 | *Dicrolene kanazawai* | Ophidiidae |  | 7 | 3.56 |
|  | 253 | *Venefica procera* | Nettastomatidae |  | 20 | 3.51 |
|  | 25 | *Bassozetus robustus* | Ophidiidae | Robust assfish | 7 | 3.71 |
|  | 69 | *Coryphaenoides mediterraneus* | Macrouridae | Mediterranean grenadier | 6 | 3.4 |
|  | 71 | *Coryphaenoides rudis* | Macrouridae | Rudis rattail | 5 | 4.5 |
|  | 200 | *Porogadus catena* | Ophidiidae |  | 6 | 3.51 |
|  | 234 | *Sternoptyx pseudobscura* | Sternoptychidae | Highlight hatchetfish | 4 | 3.39 |
|  | 4 | *Aldrovandia gracilis* | Halosauridae |  | 20 | 3.32 |
|  |  |  |  |  |  |  |
| LS-A | 24 | *Bassozetus normalis* | Ophidiidae |  | 6 | 3.63 |
|  | 1 | *Acanthonus armatus* | Ophidiidae | Bony-eared assfish | 7 | 3.62 |
|  | 32 | *Bathypterois grallator* | Ipnopidae | Tripodfish | 5 | 3.1 |
|  | 37 | *Bathysaurus mollis* | Synodontidae | Highfin lizardfish | 3 | 4.5 |
|  | 135 | *Ipnops murrayi* | Ipnopidae |  | 7 | 3.11 |
|  | 21 | *Barathrodemus manatinus* | Ophidiidae |  | 2 | 3.44 |
|  | 25 | *Bassozetus robustus* | Ophidiidae | Robust assfish | 7 | 3.71 |
|  | 30 | *Bathyonus pectoralis* | Ophidiidae |  | 3 | 3.62 |
|  | 4 | *Aldrovandia gracilis* | Halosauridae |  | 20 | 3.32 |
|  | 5 | *Alepocephalus agassizii* | Alepocephalidae | Agassiz' slickhead | 2 | 3.38 |
|  |  |  |  |  |  |  |
